# Carboxychalcones Based on Terephthalaldehydic Acid as Potential Neuroprotective Agents. Synthesis, Computational Study and Biological Evaluation

**DOI:** 10.1021/acsomega.5c01417

**Published:** 2025-05-07

**Authors:** Dorota Olender, Bartosz Skóra, Milena Kasprzak, Jacek Kujawski, Katarzyna Sowa-Kasprzak, Anna Pawełczyk, Izabela Muszalska-Kolos, Konrad A. Szychowski

**Affiliations:** † Chair and Department of Organic Chemistry, Faculty of Pharmacy, 37807 Poznan University of Medical Sciences, Rokietnicka 3, 60-806 Poznań, Poland; ‡ Department of Biotechnology and Cell Biology, Medical College, University of Information Technology and Management in Rzeszow, Sucharskiego 2, 35-225 Rzeszów, Poland; § Department of Pharmaceutical Chemistry, Faculty of Pharmacy, Poznan University of Medical Sciences, Rokietnicka 3, 60-806 Poznań, Poland

## Abstract

This work aimed to synthesize a series of carboxychalcones and determine their efficacy as neuroprotective agents in vitro. All carboxychalcones were synthesized from terephthalaldehydic acid, an aromatic aldehyde with a carboxy group, using the Claisen–Schmidt condensation method and evaluated through in silico analyses and various biological tests. The potential of chalcones derivatives bearing carboxy groups in the ring as neuroprotective agents was tested by assessing their biological effects in the in vitro model of neural cells (HT-22) inter alia, using the resazurin reduction assay, the LDH release test and flow cytometry-based methods, followed by determining the specific protein expression by Western Blot. Our research suggests that compounds **3b** and **3c** may possess antioxidant properties. In terms of the potential reactivity of the studied compounds, we discussed HOMO–LUMO descriptors using the DFT formalism and analyzed the vertical excited states (theoretical UV–vis spectra) for two compounds. The computational study proved that the relatively lowest absolute values of the HOMO–LUMO gap, electronegativity, and chemical hardness corresponded to the two derivatives tested in biological assays.

## Graphical abstract

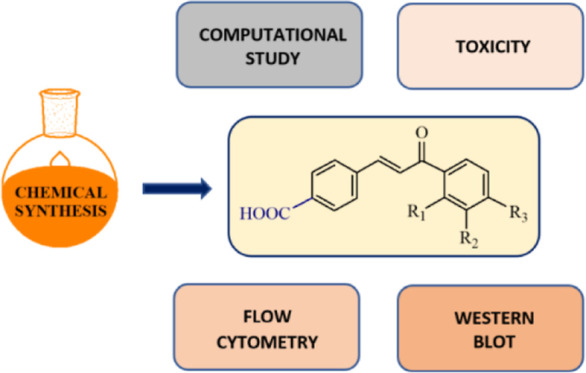

## 1 Introduction

The current challenge in medicinal chemistry is to search for new active structures with interesting and desirable biological effects. Numerous studies have revealed that it is important to know the multifaceted neuroprotective mechanisms of different compounds, including plant-derived compounds, in designing and developing novel drugs in the neuropharmacology field.^1^ Compounds belonging to the flavonoids with a chalcone structure (*E*-1,3-diphenyl-2-propen-1-one) attract particular attention and interest.^2^ The chalcone molecule is composed of two aromatic ring systems connected by an α,β-unsaturated propenone chain.^3^ These compounds are characterized by a relatively simple structure and, at the same time, a broad spectrum of biological activity, including anticancer, anti-inflammatory, antibacterial, antidiabetic, and antioxidant effects.^4–6^ Moreover, chalcones possess potential antifungal and antitubercular activities. These compounds may act as new scaffolds for the design and development of new molecules against tubercular and fungal infections.^7,8^ Both basic and suitably developed chalcones are considered in the context of candidates for new drugs dedicated to the fight against serious diseases, including Alzheimer’s (AD) and Parkinson’s disease (PD). Currently, multitarget neuroprotective agents, which are able to show protective effects, are being sought. Neuroprotectants can attenuate cerebral ischemia-induced injury, where oxidative stress and inflammation play a pivotal role in their formation.^9^ As a complementary and alternative therapy, traditional Chinese medicine, based on herbs, exerts great advantages in treating patients to improve their neurological functions, exerting mainly neuroprotective effects.^10–12^ Oxidative stress and neuroinflammation are critical factors in the development and progression of these diseases, especially AD. The combination of these two factors creates a toxic environment that accelerates neuronal loss in neurodegenerative diseases. Therefore, limiting these processes to develop potential therapeutic strategies is important, with a special focus on chalcone compounds that can mitigate these harmful effects.^13,14^

The previous works strongly show that chalcone derivatives fit very well in the role of neuroprotectants. Chalcones modulate neurotrophic, inflammatory and oxidant pathways. They show the ability to upregulate the expression of neurotrophic factors, inhibit cytokine production, and upregulate the expression of endogenous antioxidant agents, which consequently leads to the protection of CNS structures.^15^ The literature highlights recent advances in chalcone research, such as the isolation of new derivatives, innovative methods of synthesis, and evaluation of their biological properties and mechanisms of action.^4^ Chalcones offer a promising framework for the development of novel neuropharmacological agents due to their chemical versatility and wide-ranging biological activities, especially anti-inflammatory and antioxidant.^16^ Their ability to modulate multiple targets associated with neurodegenerative diseases positions them as valuable candidates in the search for effective therapies. Studies have been conducted on natural plant chalcones’ protective and antioxidant effects on brain astrocytes, highlighting oxidative stress’s role in neurodegenerative diseases.^17^ Chalcones are compounds which can scavenge free radicals and inhibit pro-inflammatory enzymes. Moreover, certain chalcone derivatives have demonstrated the ability to protect neuronal cells from damage, and also inhibit enzymes such as monoamine oxidase, acetylcholinesterase, and butyrylcholinesterase, suggesting potential in treating conditions like depression or AD and PD.^18^ The in vitro tests have been confirmed in some in vivo studies. Chalcones have shown promising neuroprotective effects on mouse hippocampal neuronal cell lines, particularly against glutamate-induced cell death or on cuprizone-induced demyelination.^19,20^ These effects are mediated through various mechanisms, including inhibition of oxidative stress, modulation of apoptotic pathways, and activation of protective signaling pathways.

Structural modifications of compounds based on the chalcone skeleton may lead to changes (increase) in the biological activity and/or the emergence of new activities. In the work conducted over the last dozen or so years, it has been found that replacing the hydroxy group with a carboxy group in the A ring of the acetophenone moiety leads to the formation of effective derivatives with higher water solubility and antibacterial activity.^21^ Carboxylic derivatives of chalcones have also been described as a new class of human immunodeficiency virus type 1 (HIV-1) integrase inhibitors.^22^ Introducing the acidic group to the B ring of the benzaldehyde moiety in the chalcone scaffold significantly increased the analgesic activity of these compounds.^23^ Chalcones with a carboxy group have also been synthesized and evaluated as effective xanthine oxidase inhibitors.^24^ These data indicate that the carboxylated B ring, linked to an alkene fragment, may be crucial for the inhibitory activity of chalcone toward this enzyme. The study results also showed that these compounds may be capable of scavenging free radicals.^24^ Moreover, the presence of carboxy and hydroxy groups in the B ring and nitrogen-containing substituents at the 4′-position or benzo nitrogen-containing heterocycles in the A ring of chalcone derivatives plays a pivotal role in potent xanthine oxidase inhibition.^25^ Furthermore, carboxylated heteroaryl-substituted chalcones have been developed as potent inhibitors of vascular cell adhesion molecule-1 (VCAM-1) expression, showing promising effects in treating chronic inflammatory diseases such as asthma.^26^ These studies highlight the diverse therapeutic potential of carboxychalcones, from pain management to anti-inflammatory applications, and underscore the importance of further research in this area. Carboxychalcones are interesting compounds that have begun to attract attention in the context of the therapy of neurodegenerative diseases of the central nervous system (CNS), such as AD, PD, and amyotrophic lateral sclerosis (ALS). Although this field is still in the research phase, some studies point to the potential benefits of carboxychalcones in treating these diseases, mainly due to their anti-inflammatory, antioxidant, and neuroprotective properties. The presence of the carboxy group can increase the molecule’s polarity and finally affect its water solubility and bioavailability.^27^ From second hand, too much increased polarity of the compound can also limit the ability to cross the blood–brain barrier, which is crucial for action in the CNS. The carboxy group can also form hydrogen bonds with different receptors or enzymes in the CNS, potentially modulating their activity. However, the crucial effect of this group on the biological activity of chalcones will depend on its position in the molecular structure and the presence of other substituents. It seems that research in the area of neurodegenerative diseases is necessary to fully understand the potential of these compounds for the treatment of neurological disorders.

This work aimed to design and synthesize carboxyl derivatives of chalcones obtained by classical alkaline Claisen–Schmidt condensation in a water–alcohol medium from appropriate aromatic aldehydes and acetophenones. The biological effect of new compounds was tested using an in vitro HT-22 neural cell model was determined, and the geometry optimization was performed using molecular docking. From the standpoint of the in silico approach, we computed the HOMO–LUMO orbitals and relevant descriptors. Cytotoxicity studies were evaluated through the resazurin reduction assay, the LDH release of the compounds was tested, and flow cytometry-based methods assay were used.

## 2 Experimental Section

### 2.1 Chemistry

#### 2.1.1 Solvents and Reagents

All reagents and solvents used in this study were purchased from commercial sources from Aldrich (Saint Louis, MO, USA), Fluka (Buchs, Switzerland), Chempur (Piekary Śląskie, Poland), and POCh S.A. (Gliwice, Poland) and directly used in the experiments. The primary antibodies against AhR (cat. 67785-1-Ig, dilution 1:3000) were kindly gifted by Proteintech (Rosemont, IL, USA). The primary antibodies against IKKβ (cat. A19606, dilution 1:2000), CASP3 (cat. A0214, dilution 1:1000), PPARγ (cat. A11183, dilution 1:1500), p­(S32)-IkBα (cat. AP0707, dilution 1:1000), IkBα (cat. A19714, dilution 1:2000), PGC-1α (cat. A20995, dilution 1:2000), GAPDH (cat. AC033, dilution 1:100,000) and NF-κB (cat. A10609, dilution 1:3000) were purchased from ABClonal (Woburn, MA, USA). The secondary HRP-conjugated-antimouse (cat. 31430, dilution 1:2500) and antirabbit (cat. 31460, dilution 1:2000) were purchased from ThermoFisher Scientific (Waltham, MA, USA).

#### 2.1.2 Instrumental Analysis

The melting points were determined on a Boetius apparatus and were uncorrected. The IR spectra were recorded using a Nicolet iS50 FT-IR spectrometer (Thermo Scientific, Waltham, Massachusetts, USA). The ^1^H and ^13^C NMR spectra were recorded using an NMR Varian VNMR-S 400 MHz spectrometer at 600, 400 (^1^H NMR) and 100 MHz (^13^C NMR), respectively (Agilent Technologies, Santa Clara, CA, USA) using tetramethylsilane (TMS) as the internal reference and DMSO-*d*_6_ as solvent. Coupling constants (*J*) are expressed in hertz (Hz). Signals are labeled as follows: s, singlet; d, doublet; dd, double doublet; m, multiplet. The MS spectra were recorded on a Bruker 320MS/420GC spectrometer apparatus (Bruker Corporation, Billerica, MA, USA) using the electron impact technique (EI), operating at 75 eV. The spectra of all compounds synthesized are available in the Supporting Information. The progress of reactions and the purity of products were checked using the TLC analysis on silica gel plates containing an ultraviolet indicator at 254 nm (DC-Alufolien Kieselgel 60 F254 from Merck, Darmstadt, Germany). Hexane and ethyl acetate (2:1, *v*/*v*) or chloroform and methanol (9:2, 10:1, *v*/*v*) were used as the eluents. The TLC spots on the plates were observed in UV light (λ = 254 nm). Silica gel 60 (63–200 μm particle size, Merck) was used for the flash column chromatography. The crude reaction products were purified using a crystallization process or flash column chromatography using hexane and ethyl acetate (2:1, *v*/*v*) or chloroform and methanol (9:2, *v*/*v*). For peak purity determination high-performance liquid chromatography were used (HPLC Agilent 1220 Infinity LC, Böblingen, Germany), a flow rate (1.0 mL/min) with a C_18_ column (Luna, Phenomenex, Shim-Pol A.M. Borzymowski, Izabelin, Poland) and with a Diode Array Detector (DAD). A mixture of acetic acid (20 mM) and KCl (1 mM)–acetonitrile (60:40 and 50:50) was used as the mobile phase. The purity of compounds detected by HPLC was higher than 95%.

#### 2.1.3 General Synthesis Procedure for Carboxychalcones **3a**–**d**

The aqueous solution of 40% NaOH (5 mL) was added dropwise to a solution of 3 mM of the appropriate aromatic methylketone (acetophenone, apocynin, paeonol, piceol) in ethanol (20 mL), and the resultant mixture was stirred with ice bath cooling. A total of 3 mM of terephthalaldehydic acid in an ethanolic solution (10 mL) was introduced into the mixture. The mixture was stirred at room temperature for 24–48 h. The mixture was then poured into ice water and neutralized with 10% HCl to produce precipitates. The solid precipitates were filtered and washed with water. The crude solid was purified by crystallization from methanol or by column chromatography using chloroform and methanol (CHCl_3_/MeOH, 9:2, *v*/*v*) as an eluent to yield the final compounds.

##### 2.1.3.1 1*E*-Phenyl-3-(4-carboxyphenyl)­prop-2-en-1-one (**3a**)

Yield: 86%, mp 225–226 °C (lit.^28^ 224–225.5 °C), *R*_f_ = 0.48 (CHCl_3_/MeOH, 9:2, *v*/*v*); FT-IR (ν, cm^–1^): 3061 (COOH), 1686 (C=O), 1609 (C=O), 1595 (C=C), 1578 (C=C), 1270, 1222, 1180, 1107, 980 (=C–H), 856, 775, 699; EI-MS, *m*/*z* (%): 252 M^+^ (38), 251 (19), 207 (60), 179 (23), 151 (3), 129 (10), 105 (47); 77 (100); ^1^H NMR (400 MHz, DMSO-*d*_6_, δ­[ppm]): 10.07 (br s, 1H, COOH), 8.10–8.08 (d, *J* = 8.80 Hz, 2H, ArH), 8.05–7.90 (m, 4H, ArH), 7.73–7.69 (d, *J* = 15.60 Hz, 1H, CH=), 7.28–7.26 (d, *J* = 8.30 Hz, 2H, ArH), 6.93–6.91 (d, *J* = 8.80 Hz, 1H, CH=), 6.77–6.74 (d, *J* = 8.80 Hz, 1H, ArH); ^13^C NMR (DMSO-*d*_6_, δ­[ppm]): 196.41 (COOH), 187.37 (C=O), 168.29, 163.14, 141.95 (CH=), 138.97, 131.66, 130.13, 129.33, 129.00, 124.31 (CH=), 115.95.

##### 2.1.3.2 1*E*-(4′-Hydroxy-3′-methoxyphenyl)-3-(4-carboxyphenyl)­prop-2-en-1-one **(3b)**

Yield: 82%, mp 241–243 °C, *R*_f_ = 0.38 (CHCl_3_/MeOH, 10:1, *v*/*v*); FT-IR (ν, cm^–1^): 3529 (OH), 3060 (COOH), 2949 (C–H), 2915 (C–H), 2849 (C–H), 1686 (C=O), 1650 (C=O), 1607 (C=C), 1577 (C=C), 1510 (C=C), 955 (=C–H); EI-MS, *m*/*z* (%): 298 M^+^ (55), 269 (12), 253 (100), 237 (28), 221 (10), 181 (10), 151 (26), 122 (5), 100 (4), 76 (4); ^1^H NMR (600 MHz, DMSO-*d*_6_, δ­[ppm]): 13.13 (br s, 1H, COOH), 10.10 (br s, 1H, OH), 8.05–8.03 (d, *J* = 15.60 Hz, 1H, ArH), 8.02–7.98 (s, 3H, ArH), 7.83 (d, *J* = 2.00 Hz, 1H), 7.82 (d, *J* = 2.00 Hz, 1H, ArH), 7.74 (d, *J* = 15.60 Hz, 1H, CH=); 7.64 (d, *J* = 2.00 Hz, 1H, ArH); 6.94 (d, *J* = 8.3 Hz, 1H, CH=); 3.89 (s, 3H, OCH_3_); ^13^C NMR (DMSO-*d*_6_, δ­[ppm]): 186.91 (COOH), 166.92 (C=O), 152.18, 147.82 (CH=), 141.28, 138.91, 132.12, 129.65, 129.23, 128.72, 124.19 (CH=), 123.93, 115.03, 111.68, 55.72 (CH_3_).

##### 2.1.3.3 1*E*-(2′-Hydroxy-4′-methoxyphenyl)-3-(4-carboxyphenyl)­prop-2-en-1-one **(3c)**

Yield: 80%, mp 253–255 °C (lit.^24^ 252–254 °C), *R*_f_ = 0.46 (CHCl_3_/MeOH, 10:1, *v*/*v*); FT-IR (ν, cm^–1^): 3529 (OH), 3060 (COOH), 2949 (C–H), 2915 (C–H), 2849 (C–H), 1687 (C=O), 1637 (C=O), 1607 (C=C), 1577 (C=C), 1510 (C=C), 955 (=C–H); EI-MS, *m*/*z* (%): 298 M^+^ (44), 251 (20), 223 (100), 210 (40), 179 (52), 151 (24), 101 (3), 44 (10); ^1^H NMR (400 MHz, DMSO-*d*_6_, δ­[ppm]): 13.32 (br s, 1H, COOH), 10.12 (br s, 1H, OH); 8.32–8.30 (d, *J* = 9.00 Hz, 1H, ArH), 8.14–8.12 (d, *J* = 15.50 Hz, 1H, ArH), 8.04–7.99 (m, 4H, ArH), 7.87–7.85 (d, *J* = 15.50 Hz, 1H, CH=), 6.60 (dd, *J* = 9.00, 2.10 Hz, 1H, CH=); 6.54 (d, *J* = 2.10 Hz, 1H, ArH); 3.86 (s, 3H, OCH_3_); ^13^C NMR (150 MHz, DMSO-*d*_6_, δ­[ppm]): 191.66 (COOH), 166.84 (C=O), 166.19, 165.72, 142.62 (CH=), 138.50, 132.87, 132.49, 129.67, 129.06, 123.50 (CH=), 113.92, 107.56, 100.94, 55.82 (CH_3_).

##### 2.1.3.4 1*E*-(4′-Hydroxyphenyl)-3-(4-carboxyphenyl)­prop-2-en-1-one **(3d)**

Yield: 78%, mp 277–279 °C (lit.^24^ 278–280 °C), *R*_f_ = 0.45 (CHCl_3_:MeOH, 9:2, *v*/*v*); FT-IR (ν, cm^–1^): 3335 (OH), 3050 (COOH), 2950 (C–H), 2920 (C–H), 2830 (C–H), 1686 (C=O), 1655 (C=O), 1607 (C=C), 1588 (C=C), 1513 (C=C), 1386, 1338, 1260, 1215, 1166, 1012, 977 (=C–H), 829, 773; EI-MS, *m*/*z* (%): 268 M^+^ (55), 267 (27), 223 (25), 195 (5), 165 (7), 136 (18), 121 (100), 93 (28), 77 (15), 65 (32); ^1^H NMR (400 MHz, DMSO-*d*_6_, δ­[ppm]): 13.10 (br s, 1H, COOH), 10.10 (br s, 1H, OH); 8.06–8.03 (d, *J* = 8.70 Hz, 2H, ArH), 8.00–7.98 (d, *J* = 8.2 Hz, 2H, ArH), 7.92–7.85 (m, 2H, ArH), 7.72–7.67 (dd, *J* = 14.70, 8.20 Hz, 1H, CH=), 7.27–7.25 (d, *J* = 8.2 Hz, 1H, ArH), 6.94–6.90 (d, *J* = 8.70 Hz, 1H, CH=), 6.74 (d, *J* = 8.7 Hz, 1H, ArH); ^13^C NMR (100 MHz, DMSO-*d*_6_, δ­[ppm]): 187.83 (COOH), 167.93 (C=O), 162.85, 141.77 (CH=), 138.98, 131.55, 130.81, 130.09, 129.68, 128.79, 124.97 (CH=), 115.98.

### 2.2 Biology

#### 2.2.1 Cell Culture

The mouse-derived hippocampal neuronal cell line (HT-22, MERCK, cat. SCC129) was cultured in a DMEM medium with 10% FBS, supplemented with 0.1% pent/strep (37 °C, 5% CO_2_). After reaching an 80%-confluency, the cells were collected by trypsinization and seeded at the density of 3.0 × 10^3^ cells/well, 5.0 × 10^5^ cells/dish, or 1.0 × 10^6^ cells/dish in 96-well plates, ⌀60 mm or ⌀100 mm culture dishes, respectively. After 24 h, the medium was removed and replaced with a fresh one containing appropriate concentrations of tested compounds (specified in the description of each method). The control was always cells treated with an equal vehicle (DMSO) as in the tested groups.

#### 2.2.2 Resazurin Reduction Assay and LDH Release Level

The method was chosen to determine the metabolic activity of the cells after treatment with the tested compounds and performed as described previously.^29^ In brief, cells were treated with **CHO** and **3a**–**d** in a concentration range between 1 nM to 100 μM for 24 or 48 h. After these time intervals, the medium was transferred to a fresh 96-well plate, and the released level of LDH was quantified using the LDH Cytotoxicity Kit according to the producer’s manual (Takara Bio). After 30 min, the absorbance was measured at 450 nm wavelength using a microplate reader (FilterMax F5, Molecular Devices). Simultaneously, the resazurin sodium salt solution (1% in DMEM) was added to the cells for 1 h, followed by measuring the fluorescence intensity at λ_ex._ = 570 nm and λ_em._ = 590 nm, using a microplate reader (FilterMax F5, Molecular Devices).

#### 2.2.3 Intracellular ROS Level

Briefly, cells were treated with 100 nM or 10 μM of **CHO**, **3b** and **3c** for 24 h. After this period, the medium was removed, and the cells were washed once with warm PBS to remove FBS residue. Next, the cells were collected by trypsinization and centrifuged at 300*g* for 5 min. The supernatant was removed, and the pellet was resuspended in a staining solution (25 μM of H2DCF-DA in serum-free DMEM) for 15 min at 37 °C, 5% CO_2_. After this, the intracellular ROS level was measured using an FL-1 filter in the flow cytometer (BD Acuri C6 Plus). The M1 gate shows the fluorescence intensity shift compared to that of the control.

#### 2.2.4 Cell Cycle

In brief, cells were treated with 100 nM or 10 μM of **CHO**, **3b** and **3c** for 24 h. Next, the cells were collected by trypsinization and centrifuged at 500*g* for 5 min, followed by removing the supernatant and washing the cells with PBS. Subsequently, the cells were centrifuged and fixed in ice-cold 70% ethanol (at −20 °C, 30 min). Next, the cells were centrifuged at 600*g* for 6 min, and the cells were rehydrated using cold PBS, followed by centrifugation. Lastly, the cell pellet was stained using 50 μg/mL of propidium iodide (PI) and 10 μg/mL of RNase in RT for 30 min. Subsequently, the cells were analyzed using a flow cytometer (BD Acuri C6 Plus) in the FL-2 filter. The cell population in certain cell cycle phases was quantified using G0/G1, S and G2/M gates, compared to the control cells.

#### 2.2.5 Western Blot

The Western Blot method was performed as described in the previous paper.^30^ HT-22 cells were treated with 100 nM of **CHO**, **3b**, and **3c** for 24 h. After this period, the medium was discarded, the cells were washed twice with PBS, and lysed using radioimmunoprecipitation assay (RIPA) buffer. The lysates were collected using a cell scraper. Protein concentration was measured using the Bradford method (BSA as a standard) and standardized across all samples. After this, 50 micrograms of protein were loaded onto 7.5%-acrylamide/bis­(acrylamide) gel. Electrophoresis was performed for approximately 1 h at 150 V, and 4 °C. Proteins were then electrotransferred from the gel onto a polyvinylidene fluoride (PVDF) membrane at 35 V, 4 °C for approximately 16 h. Nonspecific binding sites were blocked using 1% BSA in TBST for 1 h at room temperature (RT), followed by incubation with primary antibodies overnight at 4 °C with shaking (dilutions and catalog numbers of antibodies are provided in Subsection [Sec sec2.1.2]). After incubation, the membranes were washed three times with TBST and then incubated with specific HRP-conjugated secondary antibodies for 1 h. Membranes were subsequently washed four times with TBST. Enhanced chemiluminescence-based detection was performed and visualized using the C–DiGit Blot Scanner (Li-COR, Lincoln, NE, USA). The GAPDH was always used as a loading control. The band’s intensity was measured using GELQuantNet free software and compared to the control cells. Three independent repetitions were performed. The uncut, raw blots were presented in the Supporting Information (Figure S17).

### 2.3 Statistical Analysis

The data are presented as means with standard deviations (SD) from at least three independent experiments (*n* ≥ 3). The statistical analysis was performed using GraphPad Prism 8.0 (Statistical Module). The data marked with *, **, and *** indicate statistically significant differences compared to the control group at *p* < 0.05, *p* < 0.01 and *p* < 0.001, respectively (ANOVA followed by Dunnett’s posthoc test). Values marked with #, ## and ### represent statistically significant differences between specific groups at *p* < 0.05, *p* < 0.01 and *p* < 0.001, respectively (*t*-test).

### 2.4 Computational Details

The structures of compounds tested **3a**–**d** were initially optimized (Gaussian 16C.01 program^31^) using DFT formalism, namely: B3LYP,^32,33^ CAM-B3LYP,^34^ PW6B95D3,^35^ APFD,^36^ and M062X^37^ approaches. For HOMO–LUMO orbitals and UV–vis calculations, we applied the functional/6–311++G­(2d,3p) approximation (TD-DFT method), the integral equation formalism variant (IEFPCM), the linear response (LR) approach, and: methanol and water as the solvents. The HOMO–LUMO orbitals for the compounds were extracted with GaussView 5.0 program^38^ using checkpoint files.

## 3 Results and Discussion

### 3.1 Synthesis

This research chemical aims to functionalize chalcone’s basic structure by introducing a reactive carboxy group. The proposed carboxyl derivatives of chalcones were obtained as a result of the classical alkaline Claisen–Schmidt condensation in an aqueous–alcoholic medium from the appropriate aromatic aldehyde and different acetophenones.^6^ The synthesis strategy is illustrated in Scheme 1. A series of compounds were designed based on reactions of benzaldehyde with a carboxy group at position 4 of the aromatic ring. Terephthalaldehydic acid (**1**) and acetophenones selected, such as acetophenone (**2a**), apocynin (**2b**), 2-hydroxy-4-methoxyacetophenone (**2c**) or 4-hydroxyacetophenone (**2d**) were used to obtain a group of carboxychalcones (**3a**–**d**) with a group COOH at the A ring of the final chalcones (Scheme 1).

**Scheme 1:**
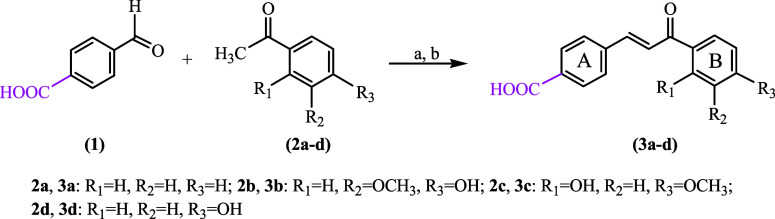
Synthesis of Carboxychalcone Derivatives **3a**–**d** ^a^

a Reagents and conditions: (a) EtOH, 40% NaOH, room temperature, 24–48 h; (b) 10% HCl.

Carboxychalcones were obtained in good yields. Compounds **3a**–**d**, derivatives of terephthalaldehydic acid, were yielded in 75–80%. The full spectral data confirmed the correct synthesis of four carboxychalcones **3a**–**d** (Figure S1–S12 in the Supporting Information). The compound’s molecular mass was supported by the molecular ions corresponding to the values consistent with the calculated molecular weights of the derivatives. The MS spectrum of compound **3a**, as an example of the characteristic fragmentation observed for this type of compound synthesized, showed that product ions were formed by losses of 28, 45, and 77 Da. It should be noted that the elimination of the COOH group is characteristic of carboxy-substituted compounds. This loss observed produced the base peak in the case of **3b**. Interestingly, that the base peak in the case of compound **3d** was produced by the elimination of the substituted phenyl of ring B with an unsaturated carbonyl chain. This one then lost the CO group to give an ion at *m*/*z* 103. In addition, losses of carboxy substituted vinylbenzenes were observed, e.g., to yield an ion at *m*/*z* 105 for compound **3a**.

A band characteristic of the stretching vibrations of the carboxy group occurring in the alkyl chain connecting the rings was found near 3000 cm^–1^. Next, the carboxyl proton was present as a broad singlet around 13.07–13.32 ppm, and the signals of the carboxyl carbon were present in the range of 186.91–198.21 ppm. In the case of the hydroxy group in the phenol moiety, the broad bands for stretching vibrations were present next to 3500 cm^–1^, and its protons were visible as singlets at 10.10–10.34 ppm. The observed chemical shifts of the hydroxy group in compounds **3b**–**c** result from the presence of methoxy groups. The presence of the carbonyl group was confirmed by the peaks near 1686 cm^–1^ or 1655 cm^–1^ in IR spectra and the signal in the range 166.84–187.37 ppm in ^13^C NMR. It should be noted that significant chemical shifts of signals from carbon both in the carboxyl and carbonyl were observed due to the influence of substituents, i.e., hydroxy and methoxy groups present in aromatic rings. The existence of the OCH_3_ groups was confirmed by the chemical shifts near 3.86 ppm in the ^1^H NMR spectra in 3b and 3c carboxychalcones. The signals from the methoxy groups occurred at approximately 55.72 and 55.82 ppm, respectively. The essential structural fragment of chalcone is an α,β-unsaturated propenone chain. It should be observed that bands of deformation and stretching vibrations of C–H bonds confirmed the presence of alkene fragments. All hydrogen atoms of the olefinic carbon–carbon bond in the propenone chain appeared as two doublets in the ranges of 7.87–7.68 ppm (Hβ) and 6.94–6.60 ppm (Hα), according to an earlier conducted study. The coupling constants (*J*) characterized high values (approximately 15.60 Hz) indicate that the obtained compounds exist as trans isomers.

### 3.2 Computational Studies

The molecular orbital frontier theory developed by Kenichi Fukui plays a key role in understanding chemical reactivity. In our paper, we used the TD-DFT method to investigate the spectra of analytes **3a**–**d**. Their geometry was previously optimized (the 6–31G­(d,p) basis set in vacuo or the presence of solvents: methanol and water). The vertical excited states were calculated using the 6–311++G­(2d,3p) basis set in methanol and water (IEFPCM). Using water as the solvent for the simulation of the cell environment for compounds **3a**–**d**, we calculated HOMO–LUMO several descriptors (Tables S1–S5 in the Supporting Information), i.e., electronegativity (χ), chemical hardness (η), and electronic potential, first ionization potential (I), and electron affinity (A) using Koopman’s theorem^39^ (Figures 1 and 2).

**Figure 1:**
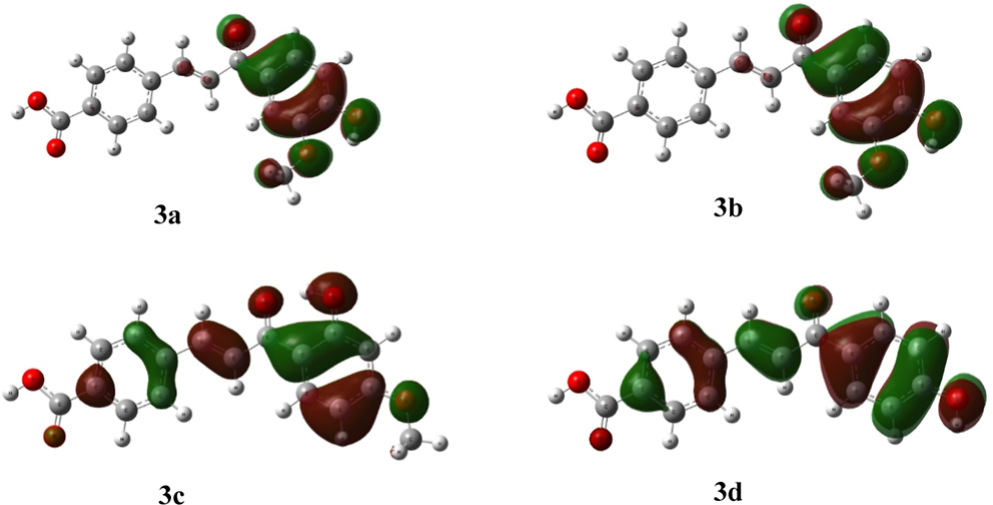
HOMO orbitals for compound compounds **3a**–**d** (optimized at the B3LYP/6–311++G­(2d,3p)//B3LYP/6–31G­(d,p) level of theory in methanol).

**Figure 2:**
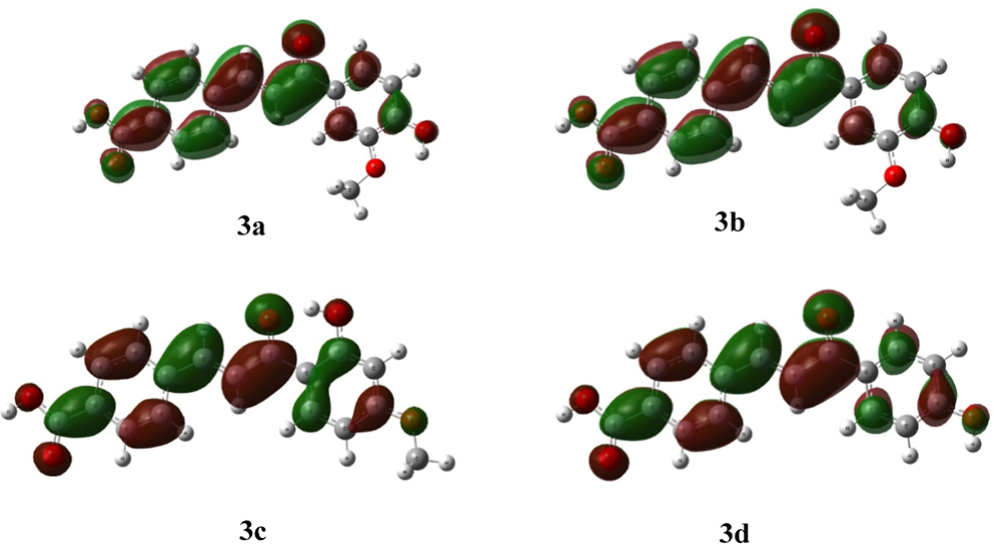
LUMO orbitals for compound compounds **3a**–**d** (optimized at the B3LYP/6–311++G­(2d,3p)//B3LYP/6–31G­(d,p) level of theory in methanol).

The HOMO orbitals (Figure 1) were primarily located over the A ring, and the LUMO orbitals (Figure 2) were located over the overall aromatic rings and –C=C– linkers. The observations regarding the positions of HOMO and LUMO orbitals within the chalcone structures were found to be consistent with literature data on other chalcone derivatives.^40,41^ Previous studies on HOMO–LUMO descriptor calculations have been limited to the use of a small number of functionals. To the best of our knowledge, the extensive comparative analysis conducted in this work within the framework of DFT formalism, utilizing as many as five functionals (B3LYP, CAM-B3LYP, PW6B95D3, APFD, and M062X), represents a significant contribution to the discussion on the electronic nature of chalcone derivatives. It also turned out that the HOMO–LUMO gap computed for **1**–**8** using the IEFPCM solvation model (Tables S1–S5 in the Supporting Information) equaled [eV]: 3.41, 5.98, 3.95, 3.62, and 5.65 (for **3a**, B3LYP, CAM-B3LYP, PW6B95D3, APFD, and M062X functionals, respectively), 3.41, 5.98, 3.95, 3.62, and 5.65 (for **3b**, B3LYP, CAM-B3LYP, PW6B95D3, APFD, and M062X functionals, respectively), 3.57, 6.11, 4.12, 3.78, and 5.78 (for **3c**, B3LYP, CAM-B3LYP, PW6B95D3, APFD, and M062X functionals, respectively), 3.78, 6.35, 4.32, 4.00, and 6.02 (for **3d**, B3LYP, CAM-B3LYP, PW6B95D3, APFD, and M062X functionals, respectively). The order of magnitude of the computed HOMO–LUMO gap and its associated descriptors were found to be comparable to the values reported in the literature for other chalcone derivatives.^40,41^ High absolute values of the HOMO–LUMO gap are generally associated with increased chemical stability and decreased reactivity. In a biological context, this often correlates with reduced biological activity, as the compound is less likely to participate in the necessary interactions with biological targets. However, it is important to note that biological activity is multifaceted and can be influenced by various factors beyond the HOMO–LUMO gap, including the presence of specific functional groups. Our computations proved that the lowest absolute values of the HOMO–LUMO gap, electronegativity, and chemical hardness mostly corresponded to derivatives **3a** and **3b**. Relatively small values of the HOMO–LUMO gap were also observed in the case of the **3c**. On the other hand, we observed that their highest theoretical absolute value was noticed for compound **3d**. On this account, we choose **3b** and **3c** to be promising in further investigations.

Additionally, within the framework of the DFT formalism (TD-DFT methodology, IEFPCM solvation model, and methanol as the solvent), we analyzed the distribution of vertical excited states for compounds **3b** and **3c**, considering the B3LYP, CAM-B3LYP, APFD, and PW6B95D3 functionals (Figure 3).

**Figure 3:**
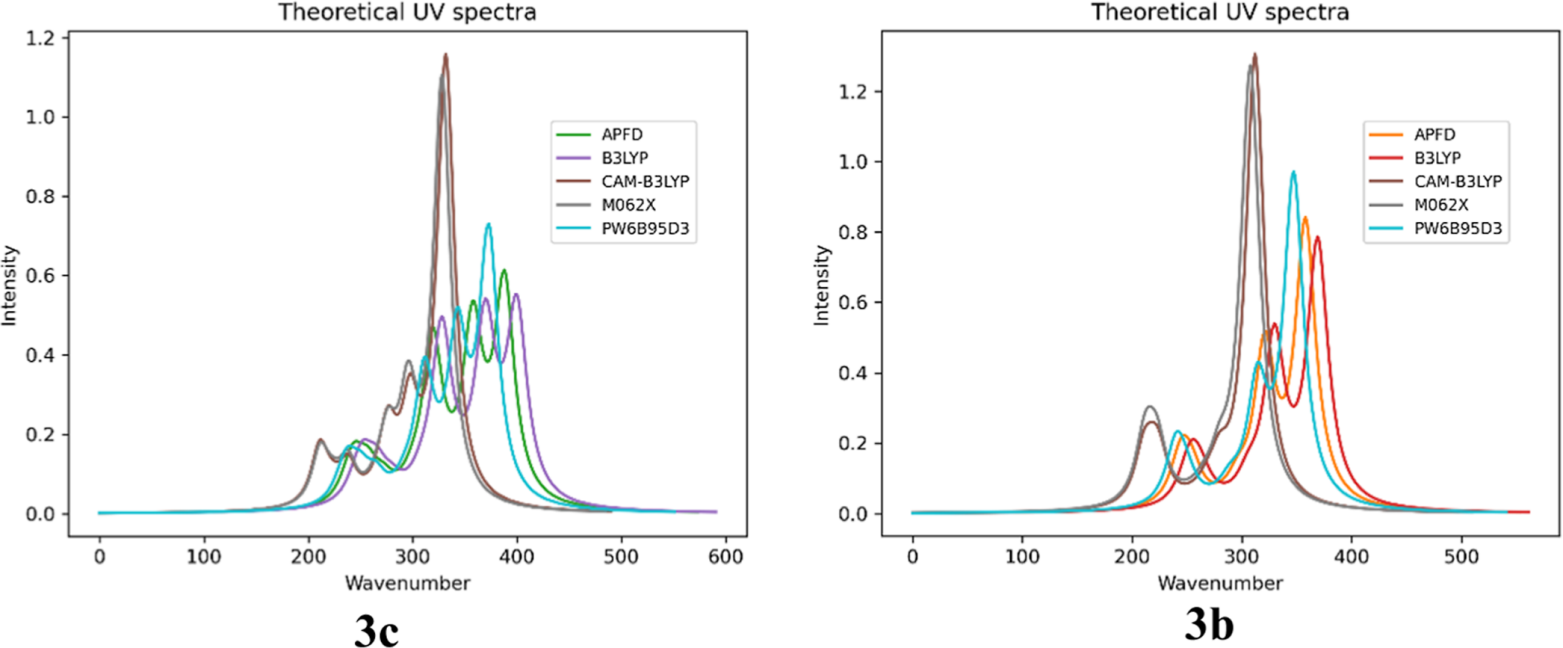
Computed UV–vis spectra of compounds **3b** and **3c** using IEFPCM solvation model and methanol as solvent; approximations: **APFD**—APF/6–311++G­(2d,3p)//APFD/6–31G­(d,p), **B3LYP**—B3LYP/6–311++G­(2d,3p)//B3LYP/6–31G­(d,p), **CAM-B3LYP**—CAM-B3LYP/6–311++G­(2d,3p)//CAM-B3LYP/6–31G­(d,p), **M062X**—M062*X*/6–311++G­(2d,3p)//M062*X*/6–31G­(d,p), **PW6B95D3**—PW6B95D3/6–311++G­(2d,3p)// PW6B95D3/6–31G­(d,p).

From the standpoint of their chemical nature and computed UV–vis spectra (Figure 3), the HOMO–LUMO gap calculated in methanol at the B3LYP/6–311++G­(2d,3p) corresponding to an electron transition from spinorbital 70 to spinorbital 71 (**3b**) or 78 to 79 (**3c**). It can be assigned to the calculated second excitation state at 368.97 nm (**3b**) or the first excitation state at 399.71 nm (**3c**). The first excited state relates mainly to the 256.31 nm (**3b**) or 252.11 nm (**3c**) bands corresponding to an electron excitation from spinorbital 68 to spinorbital 72 and a HOMO – 2 → LUMO + 1 transition (9th excitation state, oscillator strength *f* = 0.0705, coefficient 0.50959, calculated energy is 4.9116 eV; for **3b**) or from spinorbital 76 to spinorbital 80 and a HOMO – 2 → LUMO + 1 transition (9th excitation state, oscillator strength *f* = 0.1022, coefficient 0.66023, calculated energy is 4.9179 eV; for **3c**).

### 3.3 Biological Study

The influence of chalcones synthesized **3a**–**d** on the mouse hippocampal neuronal HT-22 cell line was studied by the resazurin reduction assay, the LDH release of the compounds tested, and using flow cytometry-based methods. Moreover, Western blot and cell cycle were performed.

#### 3.3.1 Metabolic Activity and LDH Release Level

After 24 h, HT-22 cells treated with **CHO** were characterized by no significant changes in metabolic activity (Figure 4A). On the other hand, after 48 h, only 50 μM and 100 μM concentrations of **CHO** were able to significantly decrease the metabolic activity of HT-22 cells by 15.27% and 20.85%, respectively, compared to the control (Figure 4A). After 24 h, derivative **3a** caused a decrease in the resazurin reduction in concentrations 10, 50, and 100 μM by 15.50, 17.06, and 17.13% respectively, compared to the control (Figure 4B). Interestingly, after 48 h of the HT-22 exposure to compound **3a**, no significant changes in the resazurin reduction were observed (Figure 4B). After 24 h, derivative **3b** caused a significant decrease in the metabolic activity by 12.42% and 44.10% after treatment of the cells with 50 μM and 100 μM concentrations, respectively (Figure 4C). No significant changes in this parameter were observed in the concentration range of **3b** between 1 nM and 10 μM (Figure 4C). On the other hand, after 48 h of treatment, a decrease by 13.08%, 12.30%, 21.17% and 64.56% in the metabolic activity was noted in cells treated with 1 μM, 10 μM, 50 μM and 100 μM, respectively, compared to the control (Figure 4C). After 24 h, HT-22 cells treated only with 100 μM of compound **3c** were characterized by a decrease in metabolic activity by 36.77%, compared to the control (Figure 4D). In turn, after 48 h of treatment, the cells treated with 50 μM and 100 μM of compound **3c** showed a decrease in metabolic activity by 16.93% and 55.36%, respectively, compared to the control, as observed (Figure 4D). After 24 h, the HT-22 cells treated with 10, 50, and 100 μM of derivative **3d** were characterized by a decrease in the resazurin reduction by 26.03, 27.89, and 31.10% respectively, compared to the control (Figure 4E). In turn, after 48 h, these cells did not show any significant changes in the resazurin reduction level after treatment with any concentration of compound **3d** (Figure 4E).

**Figure 4:**
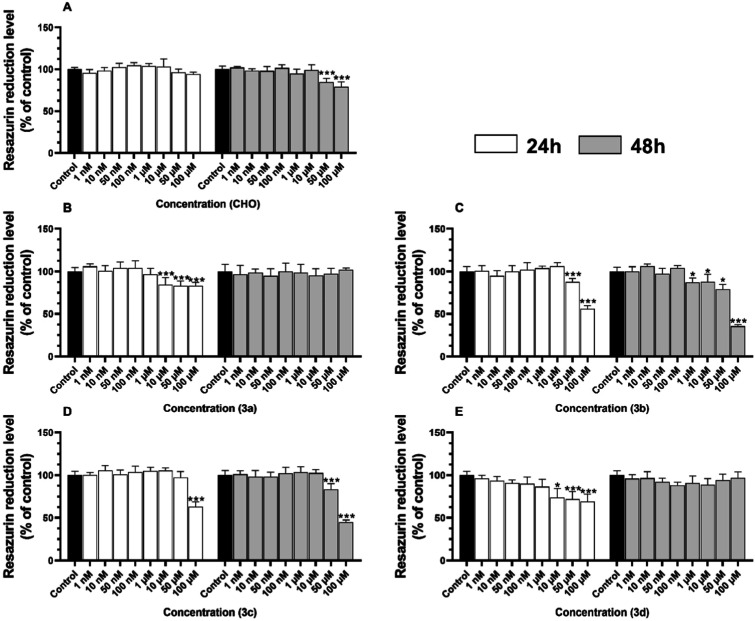
Metabolic activity after treatment of the HT-22 cells with certain concentrations of **CHO** (A), **3a** (B), **3b** (C), **3c** (D), and **3d** (E) for 24 and 48 h. The means ± SD denoted as *, **, and *** are statistically different compared to the control at *p* < 0.05, *p* < 0.01 and *p* < 0.001, respectively.

The LDH release level was not changed significantly in any tested concentrations of **CHO** after treatment for 24 nor 48 h (Figure 5A). After 24 h, compound **3a** did not significantly affect the LDH release level in HT-22 cells, compared to the control (Figure 5B). On the other hand, after 48 h, the HT-22 cells exposed to 100 μM of compound **3a** were characterized by an increase in the LDH release level by 8.86%, compared to the control (Figure 5B). On the other hand, compound **3b** in 100 μM concentration caused a significant increase in the released LDH level by 19.62% and 29.35%, compared to the control, respectively, for 24 and 48 h treatments (Figure 5C). On the contrary, after the 24 h treatment, the HT-22 cells with any concentration of **3c** did not cause any changes in the released LDH level, compared to the control (Figure 5D). In contrast, an increase in this parameter was observed after 48 h treatment of the cells with 100 μM of compound **3c** by 30.30%, compared to the control (Figure 5E). The compound **3d** in 100 μM concentration caused a significant increase in the released LDH level by 17.24% and 12.10%, compared to the control, respectively, for 24 and 48 h treatments (Figure 5E).

**Figure 5:**
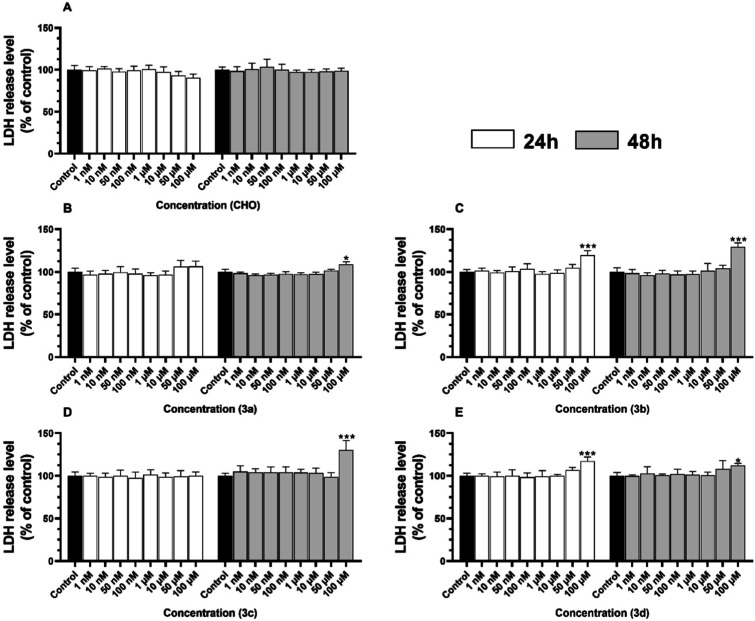
LDH release level after treatment of the HT-22 cells with certain concentrations of **CHO** (A), **3a** (B), **3b** (C), **3c** (D), and **3d** (E) for 24 and 48 h. The means ± SD denoted as *, **, and *** are statistically different compared to the control at *p* < 0.05, *p* < 0.01 and *p* < 0.001, respectively.

In the biological part, the resazurin reduction and LDH release assay showed the time- and dose-dependent effect of the tested compounds in HT-22 cells. Toxicological studies of our substances are not available in the literature. However, the research regarding similar compounds shows that chalcones from *bis-*chalcones-type compounds exhibit similar toxicity in the HT-22 cell line.^42^ Furthermore, as shown inter alia by our group in the previous paper, this group of compounds is characterized by the anticancer properties in the human colon carcinoma (Caco-2) cell line and human melanoma cell lines MeWo and A375 as well.^43,44^ Based on this, we believe that our results are in line with the current state-of-the-art. Furthermore, research shows that natural and/or synthetic chalcones are characterized by potentially beneficial properties in brain-derived cells but may also be useful in treating certain neurodegenerative and inflammation-based diseases (such as AD and PD).^4,5^

The results of the resazurin reduction tests suggest that compounds **3a** and **3d** in a wide range of concentrations (10–100 μM) decrease cell metabolism but are relatively quickly metabolized (no effect after 48 h of exposure), which in an organism could limit their usage. Both the theoretical and experimental data guided the decision to focus on **3b** and **3c** over **3a** and **3d**. In our DFT calculations, compounds **3b**, and to a slightly lesser extent **3c**, exhibited among the smallest HOMO–LUMO gaps (along with low electronegativity and chemical hardness values), indicating higher chemical reactivity or “softness”. In contrast, **3d** showed the largest HOMO–LUMO gap, implying greater stability but lower reactivity. While **3a** also featured a relatively low HOMO–LUMO gap comparable to **3b**, its biological profile was less favorable. Specifically, in the resazurin reduction assay, compounds **3a** and **3d** significantly decreased HT-22 cell metabolic activity at higher concentrations (10–100 μM) but lost their effect by 48 h, suggesting rapid metabolism or clearance in vitro, which could limit their usefulness. By comparison, **3b** and **3c** did not exhibit such transient cytotoxic effects; instead, these derivatives demonstrated potential neuroprotective benefits, such as significantly reducing intracellular ROS levels in HT-22 cells at submicromolar concentrations. Taken together, the combination of (i) favorable electronic characteristics (small HOMO–LUMO gaps associated with an increased propensity for electron transfer) and (ii) promising biological activity (antioxidant and cytoprotective effects with sustained viability) identified **3b** and **3c** as the most promising candidates. Therefore, we selected compounds **3b** and **3c** for further in-depth analyses, whereas **3a** and 3d were not pursued further due to their inferior profiles in both computational reactivity descriptors and initial biological tests.

#### 3.3.2 Intracellular ROS Level and Cell Cycle Analysis

In order to further study the potential neuroprotective mechanisms of action of compounds **3b** and **3c**, we have chosen the low concentrations (100 nM and 10 μM). The HT-22 cells treated with 100 nM of **CHO** were characterized by no significant changes in the intracellular ROS level, compared to the control, while 10 μM of this compound caused an increase in this parameter by 10.13%, compared to the control (Figure 6A). On the other hand, 100 nM and 10 μM of **3b** caused a significant decrease in the intracellular ROS level by 8.43% and 15.03%, compared to the control, respectively (Figure 6A). Similarly, the cells treated with 100 nM and 10 μM of **3c** were characterized by a decrease in this parameter by 18.70% and 21.40%, respectively, compared to the control (Figure 6A). The HT-22 cells treated with 100 nM of **CHO** were characterized by a decrease in the cell population in the G0/G1 phase by 6.81%, compared to the control, while this compound caused an increase in the cell population in S and G2/M phases by 2.14% and 4.83% respectively, compared to the control (Figure 6B). Similarly, 10 μM of **CHO** caused a decrease in the G0/G1 cell population by 8.27%, compared to the control; in turn, an increase in the cell population of S and G2/M phases was observed after treatment by this concentration by 2.59% and 5.83% respectively, compared to the control (Figure 6B). The HT-22 cells treated with 10 nM of compound **3b** were characterized by a decrease in the G0/G1 cell population by 11.40%, compared to the control, while an increase of the cell population in the S and G2/M phases by 3.46% and 7.43%, respectively, compared to the control (Figure 6). The 10 μM concentration of compound **3b** caused a decrease in the cell population in the G0/G1 phase by 10.17%, compared to the control (Figure 6B). In turn, an increase in the cell population in the S and G2/M by 3.80% and 6.70%, respectively, compared to the control after treatment of the HT-22 with 10 μM of derivative **3b** (Figure 6B). The HT-22 cells treated with 100 nM of compound **3c** were characterized by a decrease in the cell population in the G0/G1 phase by 5.34%, compared to the control while an increase in the cell population in the S and G2/M by 3.26% and 2.27%, respectively, compared to the control (Figure 6B). In turn, there was a decrease in the cell population in the G0/G1 phase by 6.57% after treatment of HT-22 cells with 10 μM of compound **3c**, compared to the control (Figure 6B). An increase in the cell population in the S and G2/M phases by 3.30% and 3.50%, respectively, compared to the control, after treatment of the cells with 10 μM of compound **3c** was observed (Figure 6B). Our research shows that **CHO** slightly increases ROS production, while compounds **3b** and **3c** strongly decrease ROS production in the HT-22 cell line. Our research suggests that compounds **3b** and **3c** could have antioxidant properties. Moreover, previous studies of other teams show that compounds **3d** and **3c** inhibit xanthine oxidase, an enzyme involved in purine metabolism and a potent source of reactive oxygen species.^24^ All studied compounds (**CHO**, **3b**, and **3c**) similarly affect the cell cycle. A decrease in the G0/G1 cell population suggests an increase in the number of cells proliferating or preparing to divide.^45^ However, compound **3c** had the weakest proliferation-stimulating properties. Interestingly, studies conducted by Shudo et al. show that 1 μM compound **3a** weakly initiates the differentiation of promyelocytic leukemia cell line (HL-60) into myelocytes (5% of cells) and neutrophils (0.50% of cells).^28^ The mentioned discovery could explain why compound **3c**, to a lesser extent, stimulates cell proliferation.

**Figure 6:**
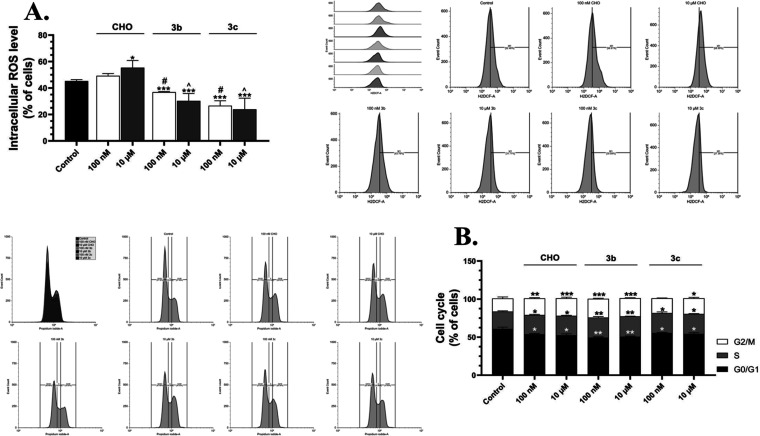
Intracellular ROS level (A) and cell cycle analysis (B) after treatment of the HT-22 cells with 100 nM and 10 μM of **CHO**, **3b** and **3c** for 24 h. The representative cytograms and overlays were shown next to the graphs. The means ± SD denoted as *, **, and *** are statistically different, compared to the control at *p* < 0.05, *p* < 0.01 and *p* < 0.001, respectively. The data denoted as # shows significant differences between 100 nM of compound **3b** or **3c**, compared to the 100 nM of **CHO** at *p* < 0.05, while data denoted as ^ shows significant differences between 10 μM of compound **3b** and **3c**, compared to the 10 μM of **CHO** at *p* < 0.05.

#### 3.3.3 Protein Expression

In the final part of our study, we determined the expression of specific proteins to evaluate the impact of the obtained compounds at the proteome level. The protein expression of IKKβ was not changed in cells treated with any of the tested compounds (Figure 7A). In turn, the expression of pro-CASP3 was decreased by 9.48% and 14.22%, compared to the control, respectively for the HT-22 exposed to the **CHO** and compound **3c** (Figure 7B). On the other hand, no significant changes were observed at the cleaved-CASP3 protein level in any of the tested compounds (Figure 7C). A decrease in activation of CASP3, which may suggest the antiapoptotic effect of these two substances. Additionally, the (AhR) protein expression was decreased only in the HT-22 cells treated with **CHO** by 8.24%, compared to the control, while compounds **3b** and **3c** did not cause any changes in this parameter (Figure 7D). Similarly, the peroxisome proliferator-activated receptor gamma (PPARγ) protein expression was decreased only in cells treated with **CHO** by 34.62%, compared to the control (Figure 7E). Moreover, the phosphorylation level of the IkBα was increased in cells treated with **3c** by 51.48%, compared to the control (Figure 7F). Conversely, a proportional decrease in the pan-IkBα protein expression by 24.62%, compared to the control was observed in cells treated with **3c** (Figure 7G). On the other hand, no significant changes in the peroxisome proliferator-activated receptor-gamma coactivator-1 alpha (PGC-1α) protein expression were observed in any of the tested compounds (Figure 7H). However, cells treated with **3c** were characterized by an increase in the NF-κB protein expression by 46.79%, compared to the control (Figure 7I).

**Figure 7:**
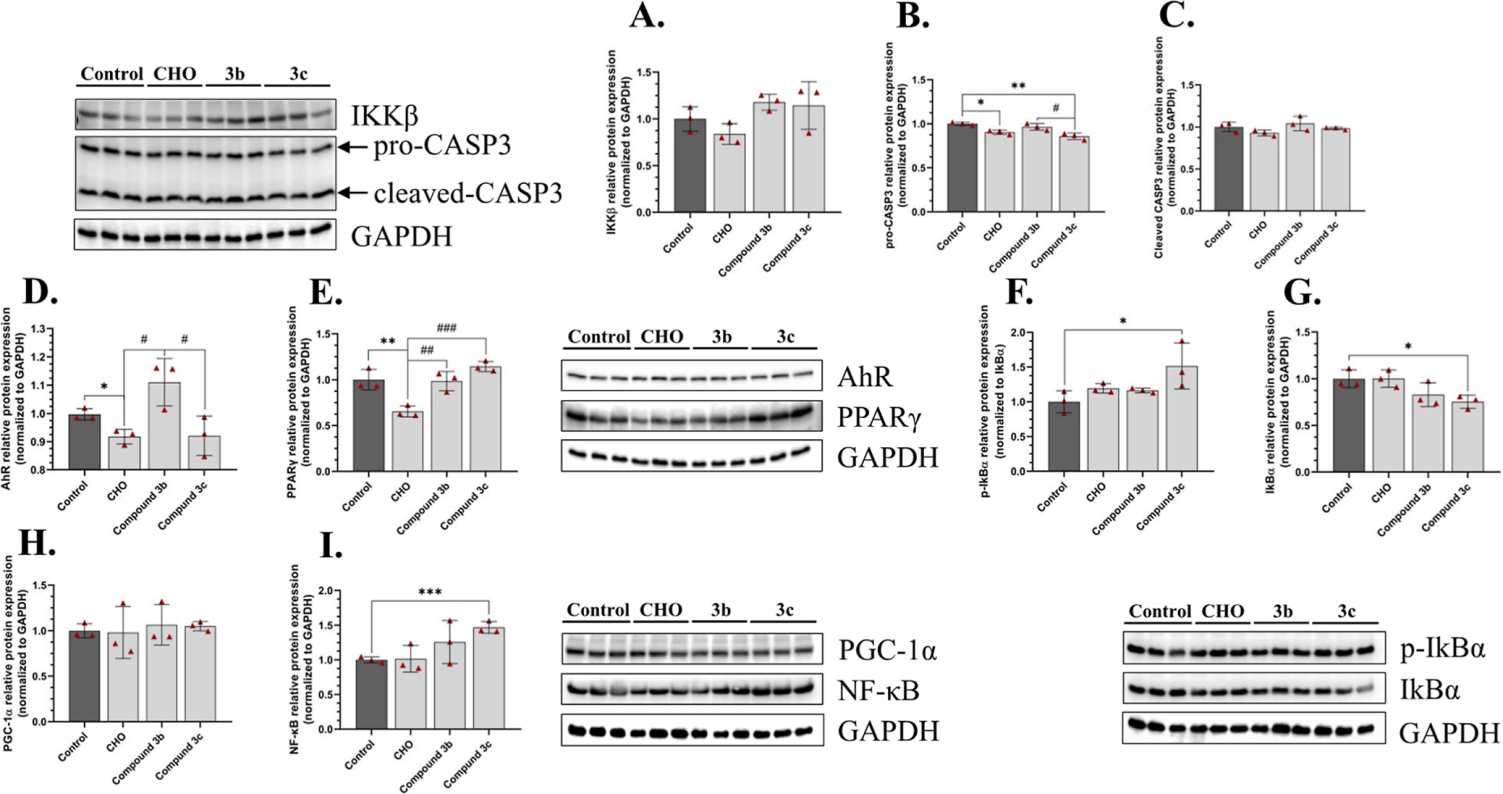
Protein expression of IKKβ (A), pro-CASP3 (B), cleaved CASP3 (C), AhR (D), PPARγ (E), p-IkBα (F), pan-IkBα (G), PGC-1α (H) and NF-κB (I) after treatment of the HT-22 cells with DMSO (Control), 100 nM of **CHO**, **3b** or **3c** for 24 h. The means ± SD denoted as ***, **, and * are statistically different, compared to the control by *p* < 0.001, *p* < 0.01, and *p* < 0.05, respectively. The data denoted as #, ## and ### is statistically different between certain groups at *p* < 0.05, *p* < 0.01 and *p* < 0.001, respectively.

Subsequent studies of AhR, PPARγ and PGC-1α protein levels suggest that compounds **CHO** and **3c** interact with the AhR while only **CHO** significantly affects PPARγ expression. To date, it is well described that both AhR and PPARγ are receptors for xenobiotics and environmental factors and can be blocked or activated by flavonoid derivatives.^46,47^ Therefore, we can assume that the studied chemical compounds have affinities for the mentioned receptors. The main function of PPARγ during the inflammatory reaction is to promote the inactivation of NF-κB, which is the main factor regulating this process.^48^

The nuclear factor kappa-light-chain-enhancer of activated B cells (NF-κB) is one of the most important regulators of proinflammatory gene expression.^14,49^ While the nuclear factor of kappa light polypeptide gene enhancer in B-cells inhibitor alpha (IκBα) is a family of cellular proteins that inhibits the NF-κB transcription factor.^50^ Moreover, an increase in the level of p-IκBα with a concomitant decrease in the level of IκBα may correlate with increased inflammation.^51^ Furthermore, it has been described that the inhibitor of nuclear factor kappa B kinase subunit beta (IKKβ) participates in the activation of NF-κB and in the promotion of inflammation.^52^ In our experiments, the expression profile of IKKβ is similar to changes in the NF-κB expression profile. However, changes in IKKβ expression were not statistically significant. Based on our results, we can assume that compound **3c** potentially initiates the inflammation process in the HT-22 cells. Compound **3b** also affects the expression of NF-κB, IκBα and p-IκBα.

Comparing the influence of the structure of the obtained chalcones on the toxicity of the HT-22 cells, it should be noted that the compound without substituents reduced the resazurin level only at the highest concentrations after 48 h. The introduction of a carboxy group in ring A (**3a**) caused a decrease in the resazurin reduction test after 24 h at the highest tested concentrations of this compound (10–100 μM). Analogues toxicity results were observed for compound **3d**, which contained, in addition to the COOH group in ring A, also the OH group in ring B. Interestingly, after 48 h exposure of the HT-22 cells to compounds **3a** and **3d**, no significant changes were observed in the cells. However, the introduction of these groups increased the release of LDH at the highest concentrations of compounds **3a** and **3d**, while compound **CHO**, without substituents, did not cause any significant changes in this test. Introducing a carboxy group in ring A and hydroxy and methoxy groups in ring B of the chalcone reduced the resazurin reduction test and increased the release of LDH. Compound **3b**, having a hydroxyl at the 4 position and a methoxyl at the 3 position, showed toxicity at lower concentrations and a shorter test time than its isomer **3c**, containing these groups at the positions 2 and 4 of ring B, respectively. Introducing electron-donating groups into the B ring of the chalcone affected the intracellular ROS level. Chalcone **CHO**, without substituents, slightly increased ROS production, whereas isomeric compounds containing methoxy and hydroxy groups (**3b**, **3c**) significantly reduced this parameter production in the HT-22 cell lines at lower concentrations. Isomer **3c,** containing OH at the 2 position and OCH_3_ at the 4 position of the ring B was more effective. On the other hand, this compound had the weakest proliferation-stimulating properties in the G0/G1 phase of the cell cycle. Analysis of protein expression indicates that the actions of isomeric compounds may be opposite and proceed via different molecular pathways. Of the obtained isomers (**3b**, **3c**) differing in the positions of the hydroxy and methoxy groups in ring B, only compound **3c** may have affinity for the tested protein receptors. A similar effect was also demonstrated by the chalcone without substituents (**CHO**)—it interacts with AhR and significantly affects the expression of PPARγ.

The relatively high polarity of the studied carboxychalcone derivatives is an important factor influencing their central bioavailability. The presence of a carboxy group greatly increases hydrophilicity and water solubility but concomitantly reduces lipophilicity. In practical terms, anionic carboxylates have poor partitioning into the CNS, and excessive polarity is known to limit BBB permeability.^27^ Thus, although the carboxy substituent might offer favorable pharmacokinetic traits (e.g., improved systemic distribution or target binding through hydrogen-bond interactions), it may impair the compounds’ ability to reach effective concentrations in the brain. This trade-off between solubility and CNS penetration suggests that the neuroprotective efficacy observed in vitro might not directly translate in vivo without additional strategies. In the Results and Discussion, we acknowledge that the polarity imparted by the carboxy group could restrict BBB crossing, and therefore, the actual availability of these chalcones in the central nervous system could be limited. This consideration underscores the need for further studies on BBB permeability or possible prodrug modifications to ensure that compounds **3b** and **3c** can effectively reach their CNS targets.

## 4 Conclusions

A series of carboxychalcone derivatives with or without differently located electron-donating substituents (OH, OCH_3_) have been synthesized and assessed for their activity as free radical scavengers and potential neuroprotective effects. The carboxychalcones were obtained in one-pot Claisen–Schmidt reactions with good yields. A set of standard spectroscopic methods successfully confirmed the structural identities. Then, an evaluation of the cytotoxic activity potential of the compounds was performed using various assays. A low cytotoxicity effect on the HT-22 cells characterizes the compounds tested. The antioxidant character of derivatives **3b** and **3c** suggests a potentially beneficial application of these compounds in further studies. Unfortunately, our preliminary molecular studies show that compounds **3b** and **3c** may potentially initiate inflammation through PPARγ/NF-κB pathways. The order of magnitude of the computed HOMO–LUMO gap and its associated descriptors was found to be comparable to the values reported in the literature for other chalcone derivatives. Our computations proved that the relatively lowest absolute values of the HOMO–LUMO gap, electronegativity and chemical hardness corresponded to derivatives **3b** and **3c**. We also observed that their lowest theoretical absolute value was observed for compound **3d**.

Our findings highlight **3b** and **3c** as promising neuroprotective carboxychalcones with antioxidant properties in neuronal cell models. To strengthen the translational impact of these results, the next crucial steps will involve evaluating the brain availability and in vivo efficacy of these compounds. In particular, dedicated blood–brain barrier permeability assays (e.g., in vitro BBB models or parallel artificial membrane permeation tests) are warranted to determine whether **3b** and **3c** can sufficiently cross into the brain. Positive results from such experiments would pave the way for in vivo studies in appropriate animal models of neurodegeneration or oxidative stress to confirm that these chalcones can exert neuroprotective effects within a living organism. Therefore, as a final outlook, we plan to investigate the BBB penetrance of **3b** and **3c** and to perform in vivo neuroprotection studies. These future investigations will be critical to validate the therapeutic potential of compounds **3b** and **3c** and to advance them toward possible development as CNS-active agents.

## Supplementary Material



## Notes

The spectra of the carboxychalcones. Biological data of the carboxychalcones. HOMO–LUMO descriptors computations for **3a–d** (PDF)
