# Supplementary material for: Carboxychalcones Based on Terephthalaldehydic Acid as Potential Neuroprotective Agents. Synthesis, Computational Study and Biological Evaluation
Source: ACS Omega. 2025 May 7;10(19):19860–72. doi: 10.1021/acsomega.5c01417 (PMC12096237; doi:10.1021/acsomega.5c01417)
Supplement: Supplementary file 1 [file ao5c01417_si_001.pdf]

Carboxychalcones Based on Terephthalaldehydic Acid as Potential Neuroprotective Agents. Synthesis, Computational Study and Biological Evaluation

Dorota Olender[1*], Bartosz Skóra[2], Milena Kasprzak[1], Jacek Kujawski[1], Katarzyna Sowa-Kasprzak[1], Anna Pawełczyk[1], Izabela Muszalska-Kolos[3], Konrad A. Szychowski[2]

[1] Chair and Department of Organic Chemistry, Faculty of Pharmacy, Poznan University of Medical Sciences, Rokietnicka 3, 60-806 Poznań, Poland

[2] Department of Biotechnology and Cell Biology, Medical College, University of Information Technology and Management in Rzeszow, 35-225 Rzeszów, Poland

[3] Department of Pharmaceutical Chemistry, Faculty of Pharmacy, Poznan University of Medical Sciences, Rokietnicka 3, 60-806 Poznań, Poland

SPECTRA OF THE CARBOXYCHALCONES 3a-d

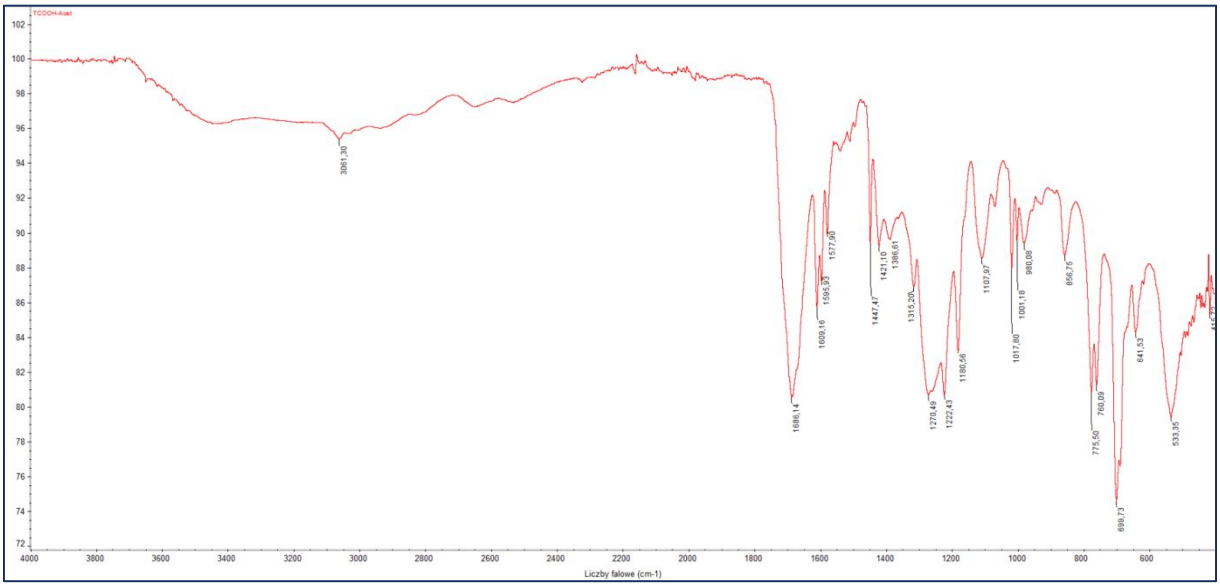

Figure S1. FT-IR spectrum of 3a

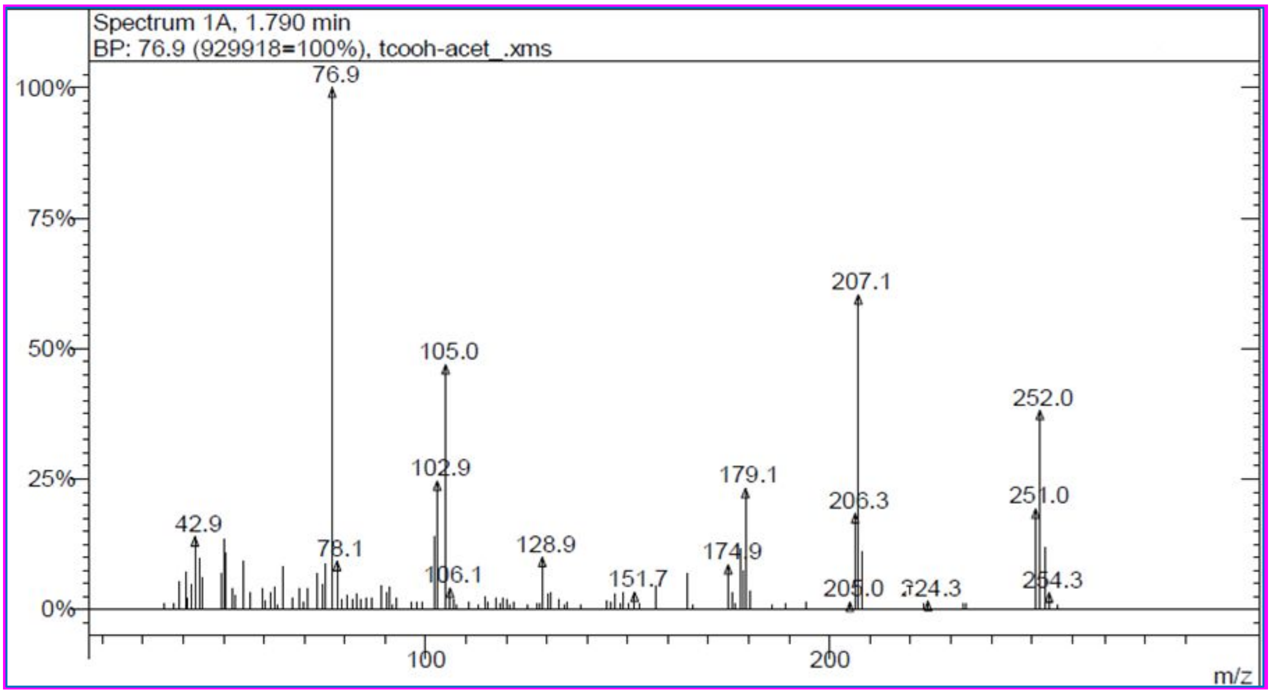

Figure S2. EI-MS spectrum of 3a

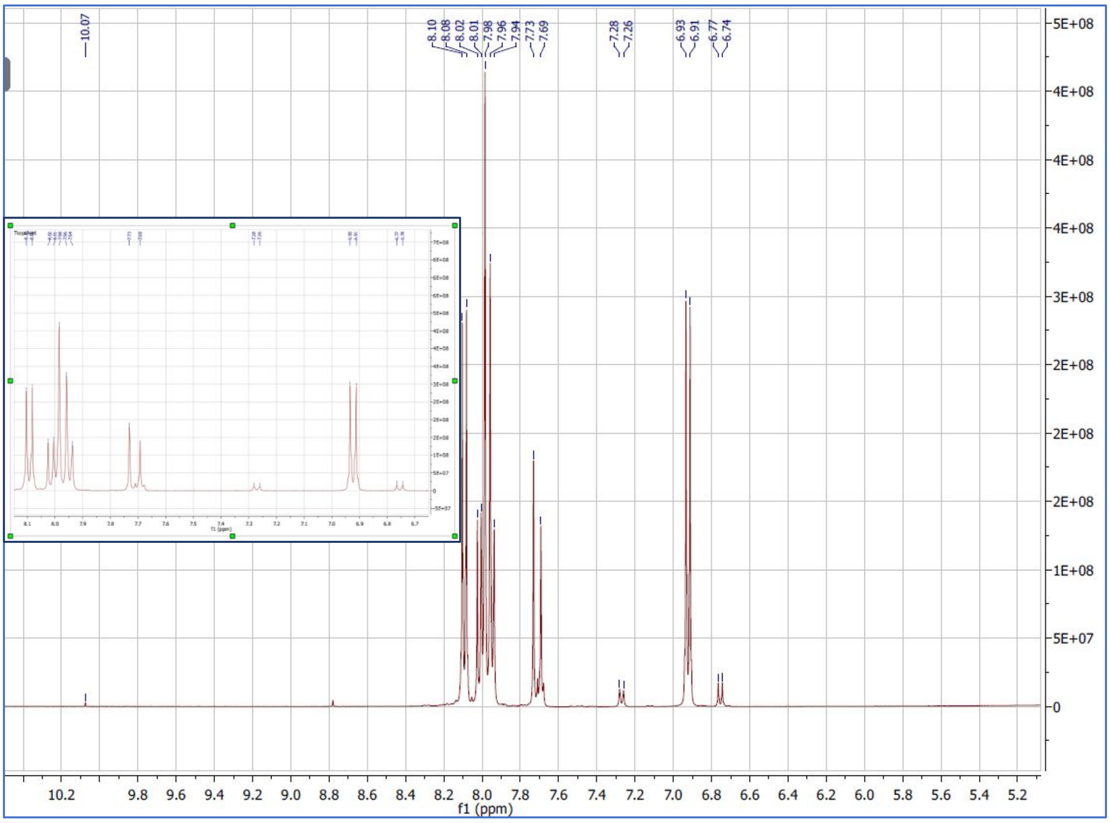

Figure S3. ^{1}H NMR spectrum of 3a

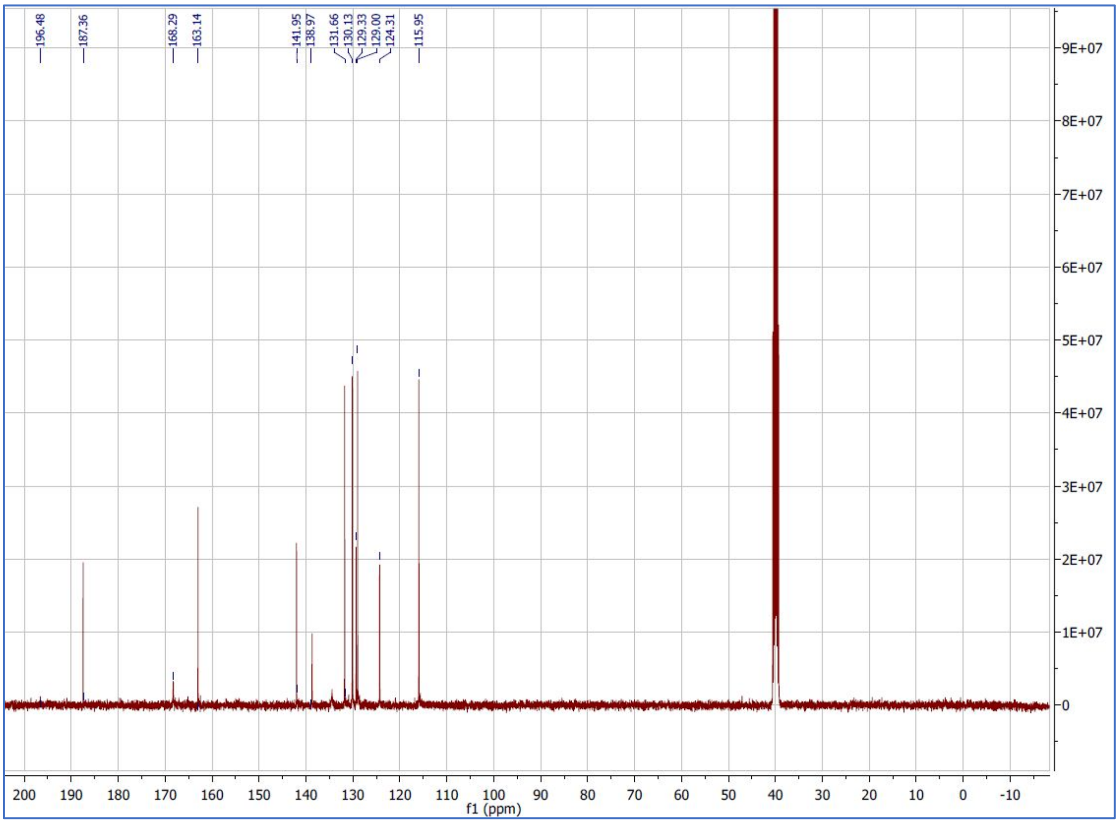

Figure S4. ^{13}C NMR spectrum of 3a

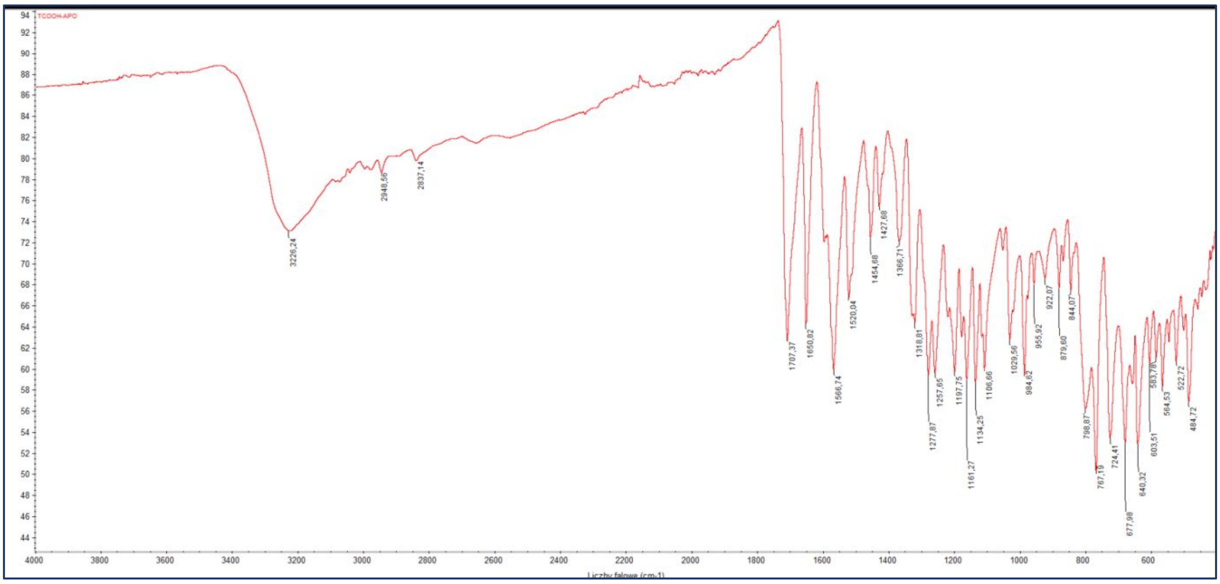

Figure S5. FT-IR spectrum of 3b

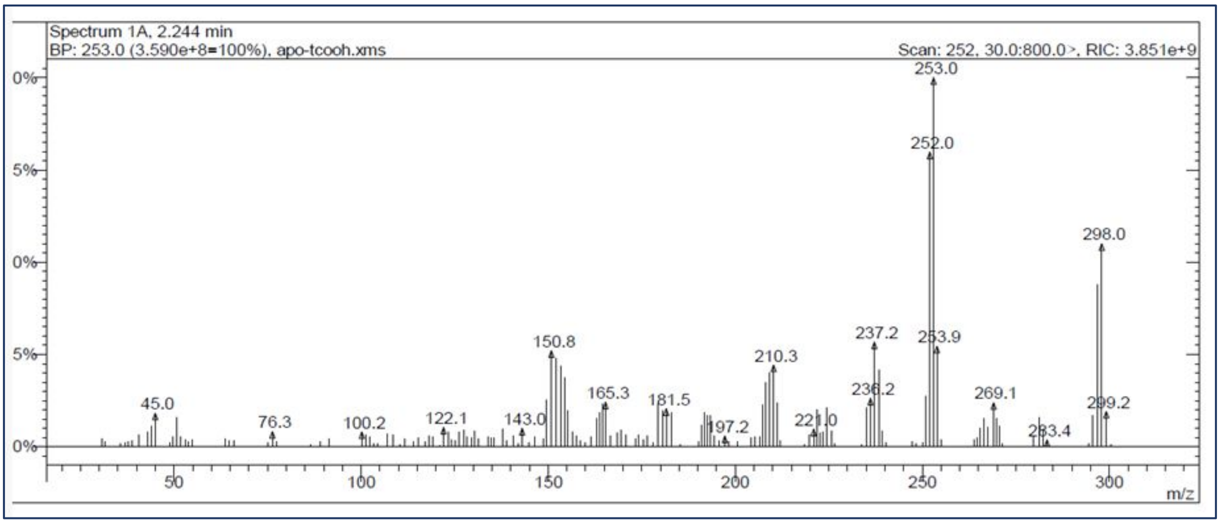

Figure S6. EI-MS spectrum of 3b

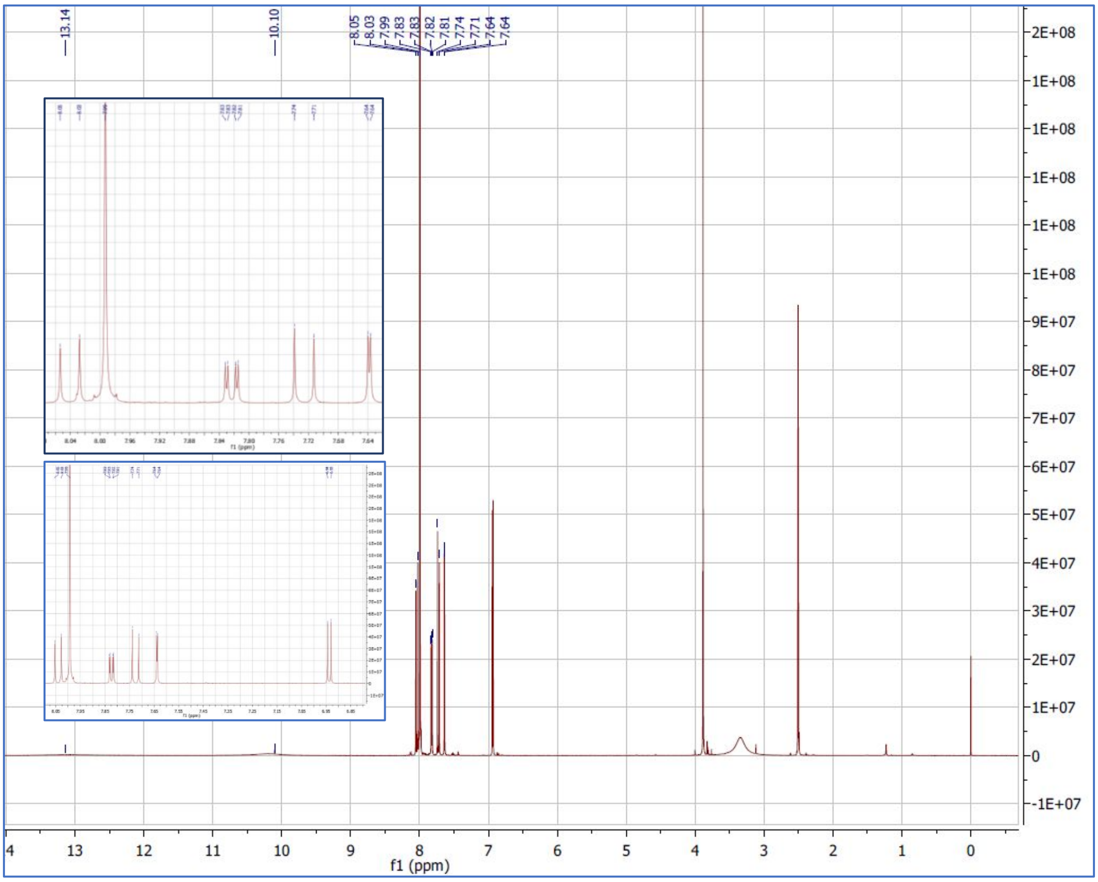

Figure S7. ^{1}H NMR spectrum of 3b

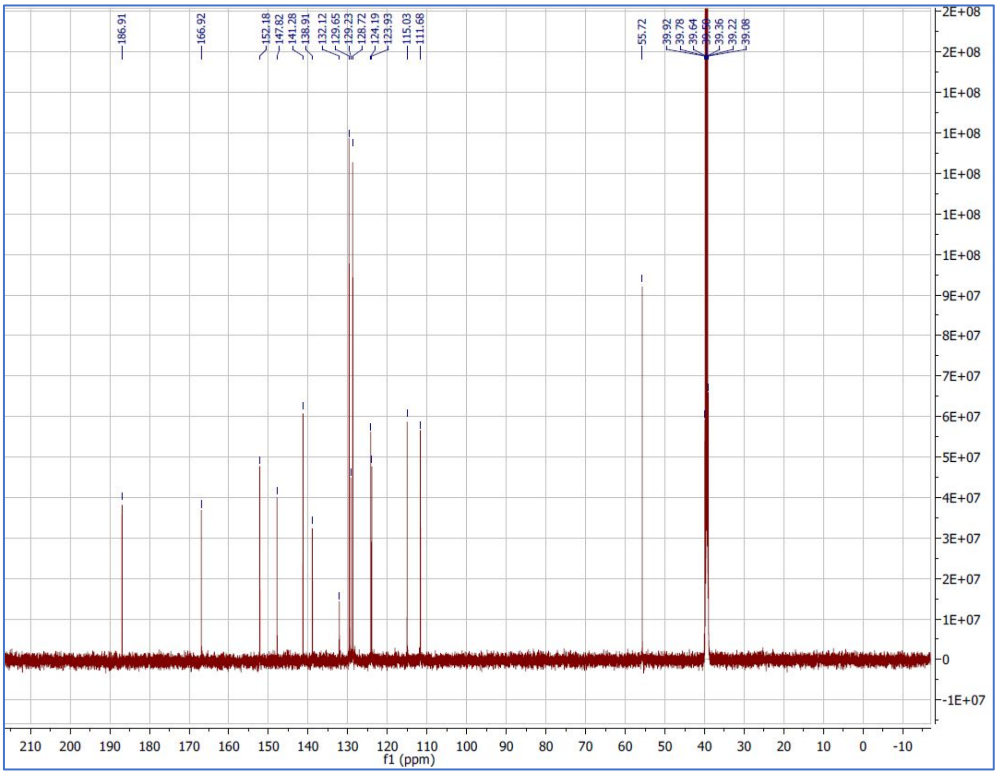

Figure S8. ^{13}C NMR spectrum of 3b

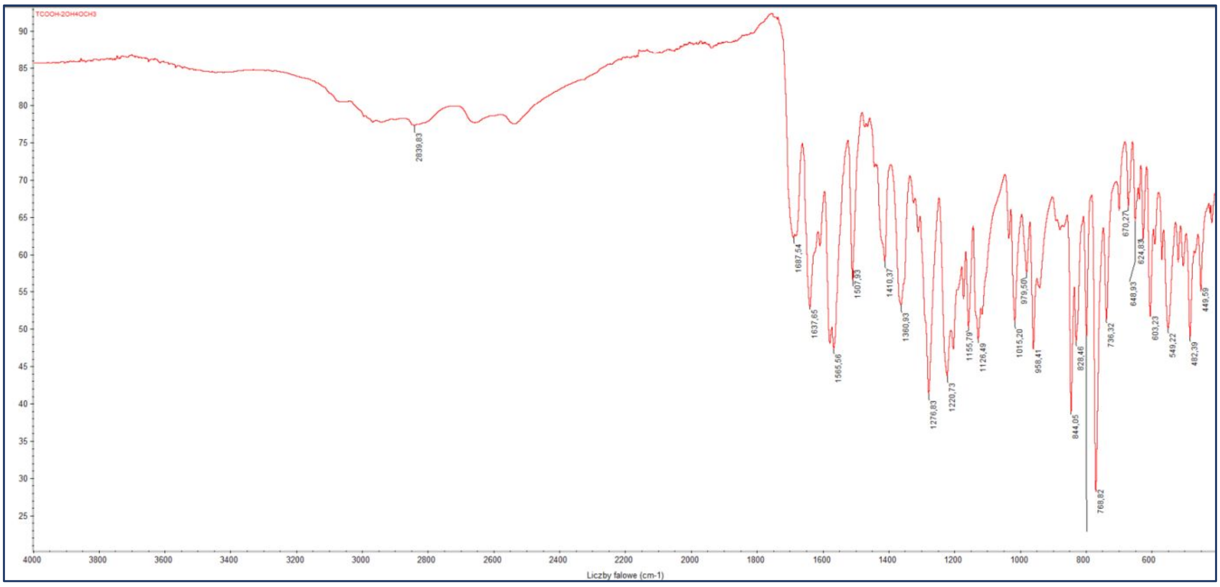

Figure S9. FT-IR spectrum of 3c

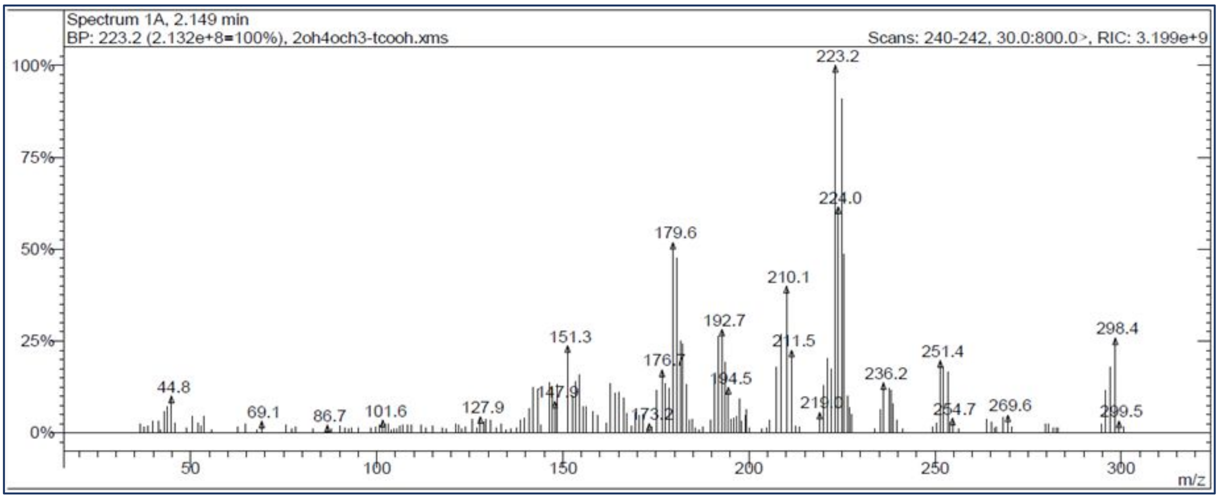

Figure S10. EI-MS spectrum of 3c

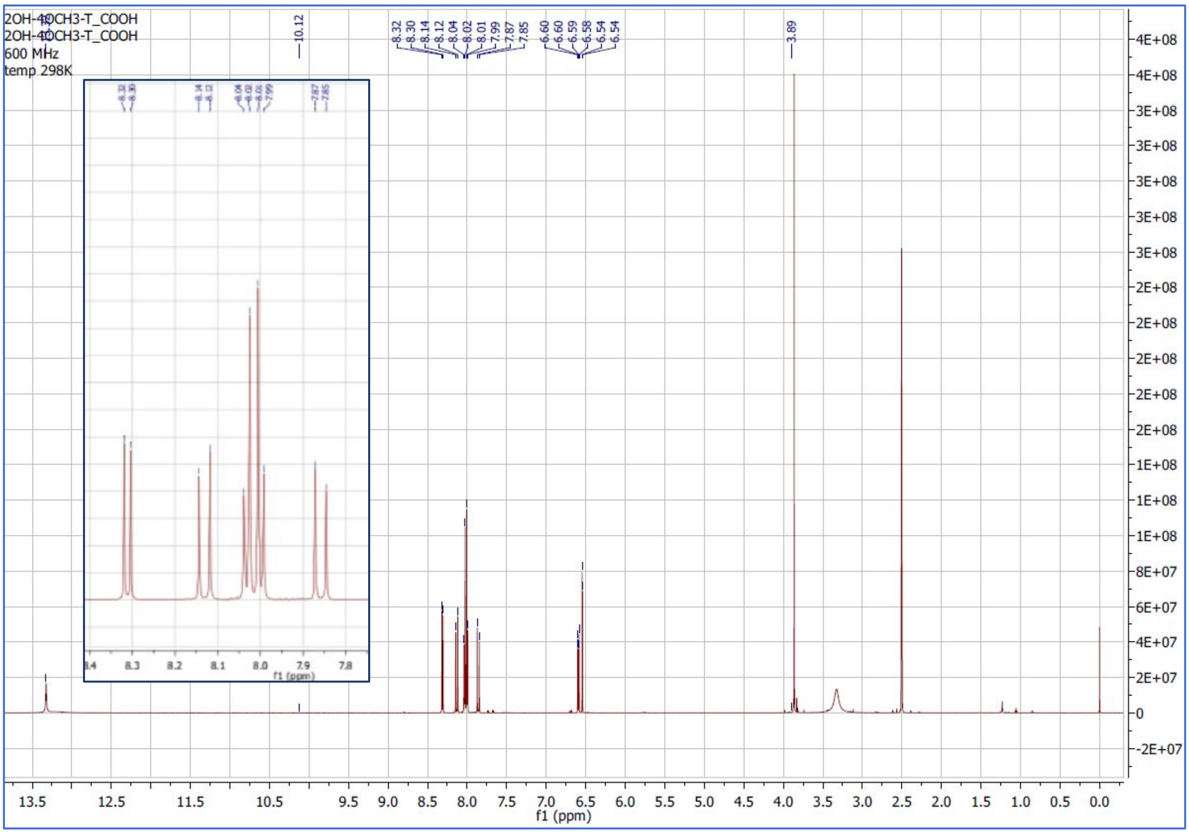

Figure S11. ^{1}H NMR spectrum of 3c

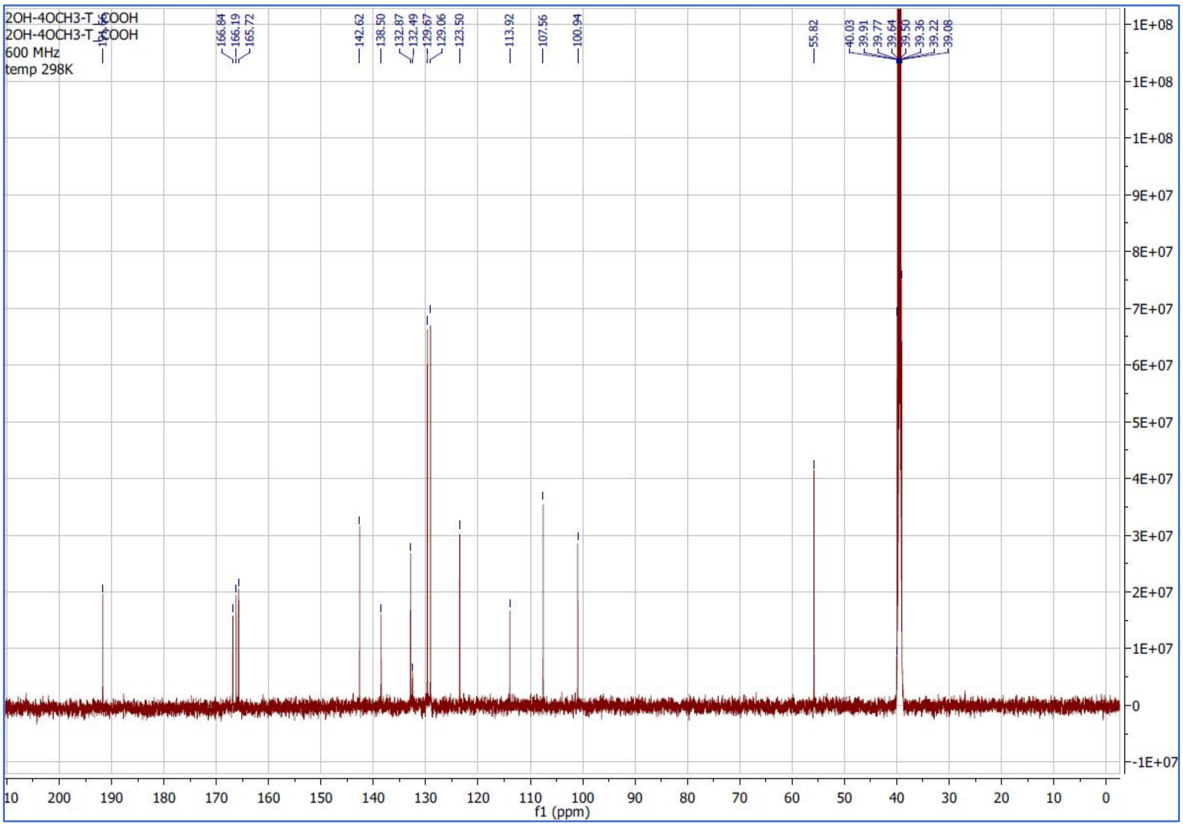

Figure S12. ^{13}C NMR spectrum of 3c

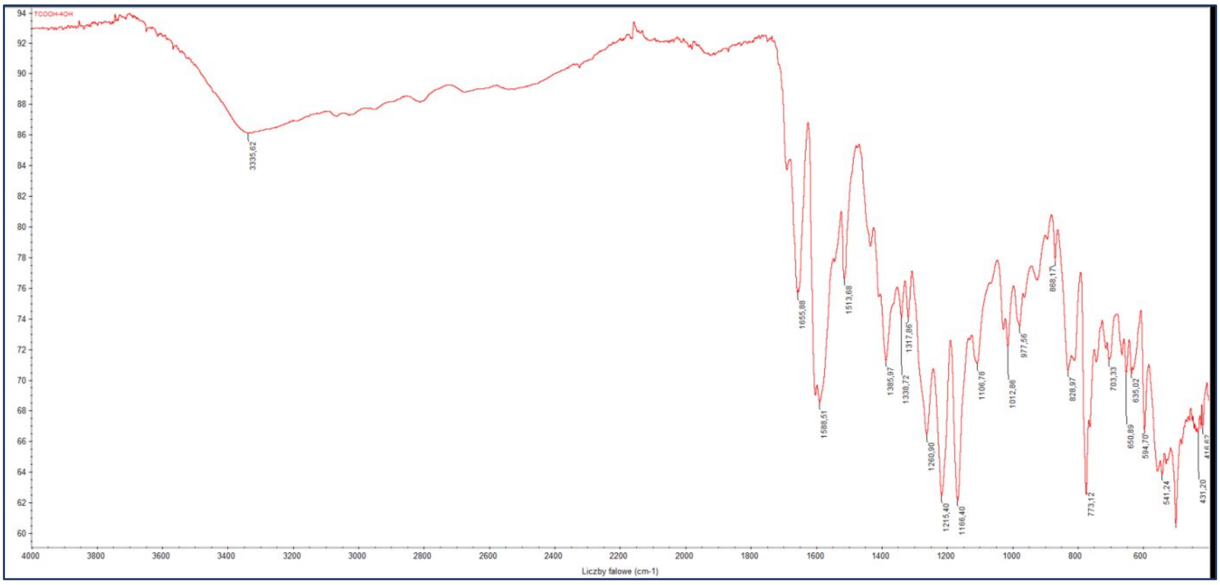

Figure S13. FT-IR spectrum of 3d

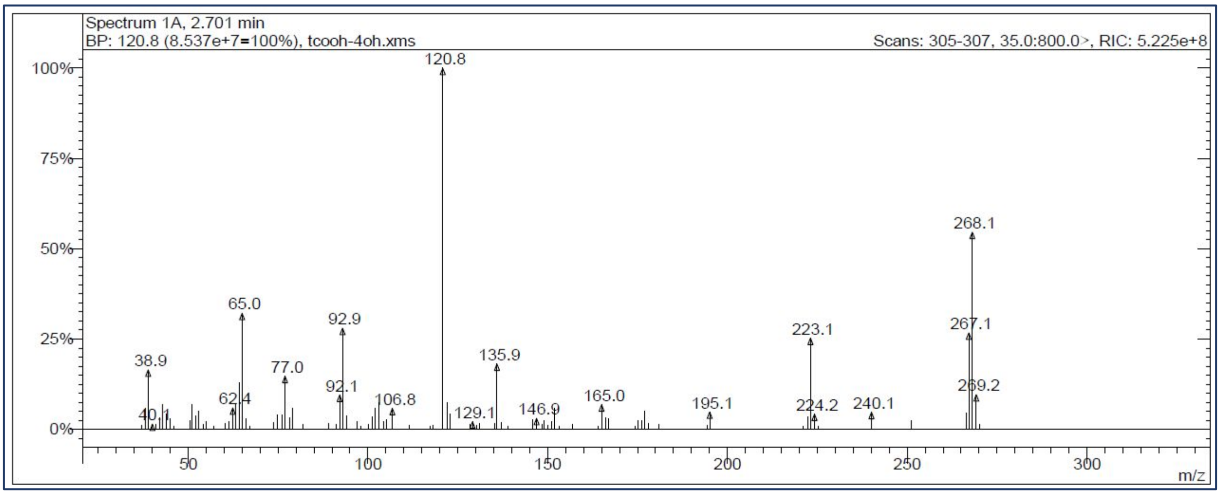

Figure S14. EI-MS spectrum of 3d

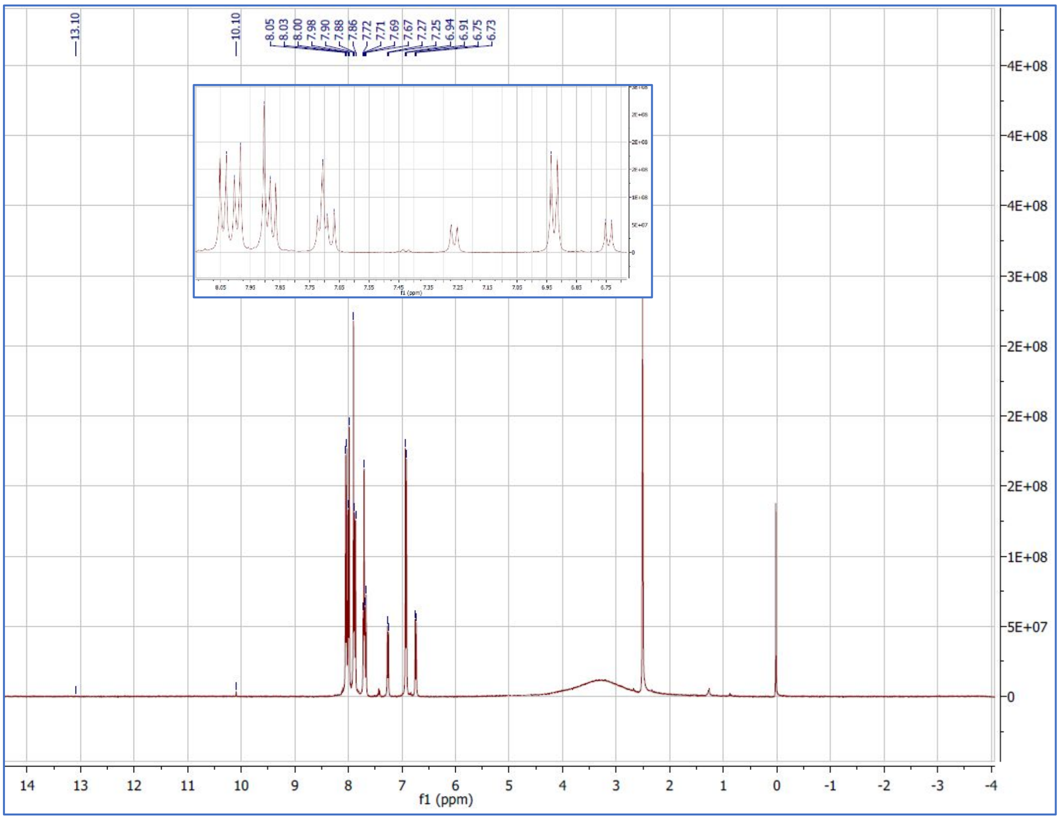

Figure S15. ^{1}H NMR spectrum of 3d

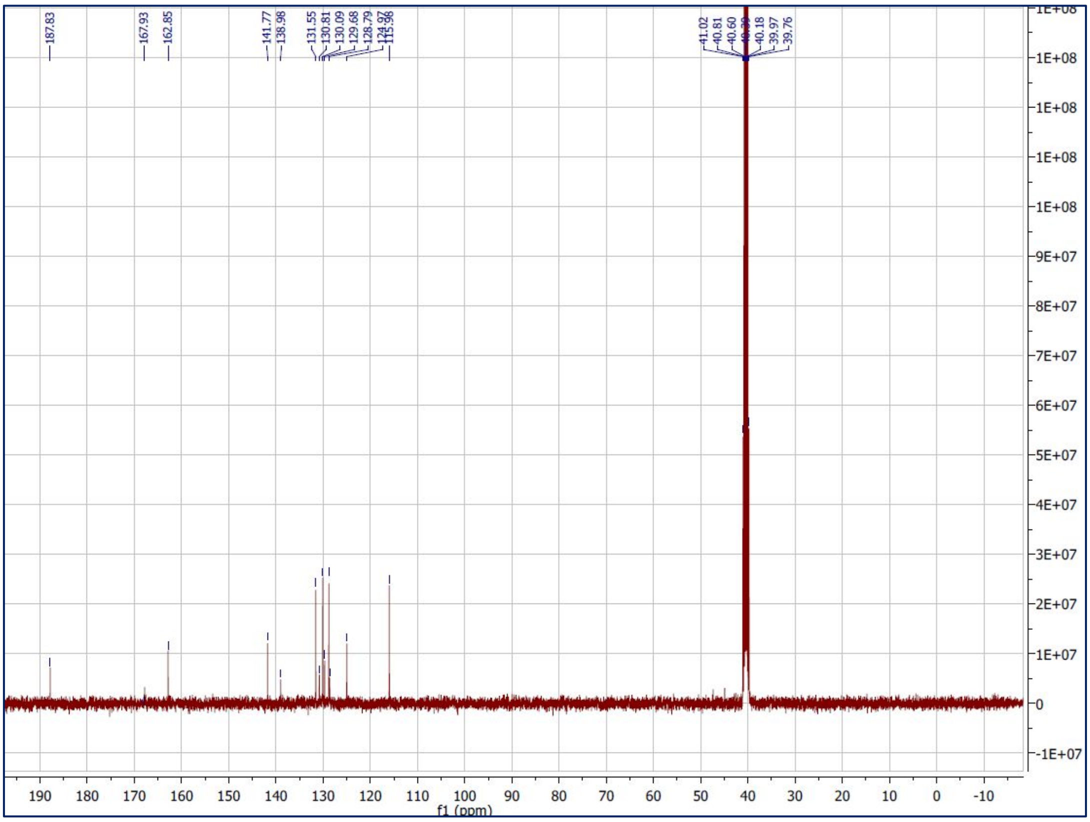

Figure S16. ^{13}C NMR spectrum of 3d

BIOLOGICAL DATA OF THE CARBOXYCHALCONES

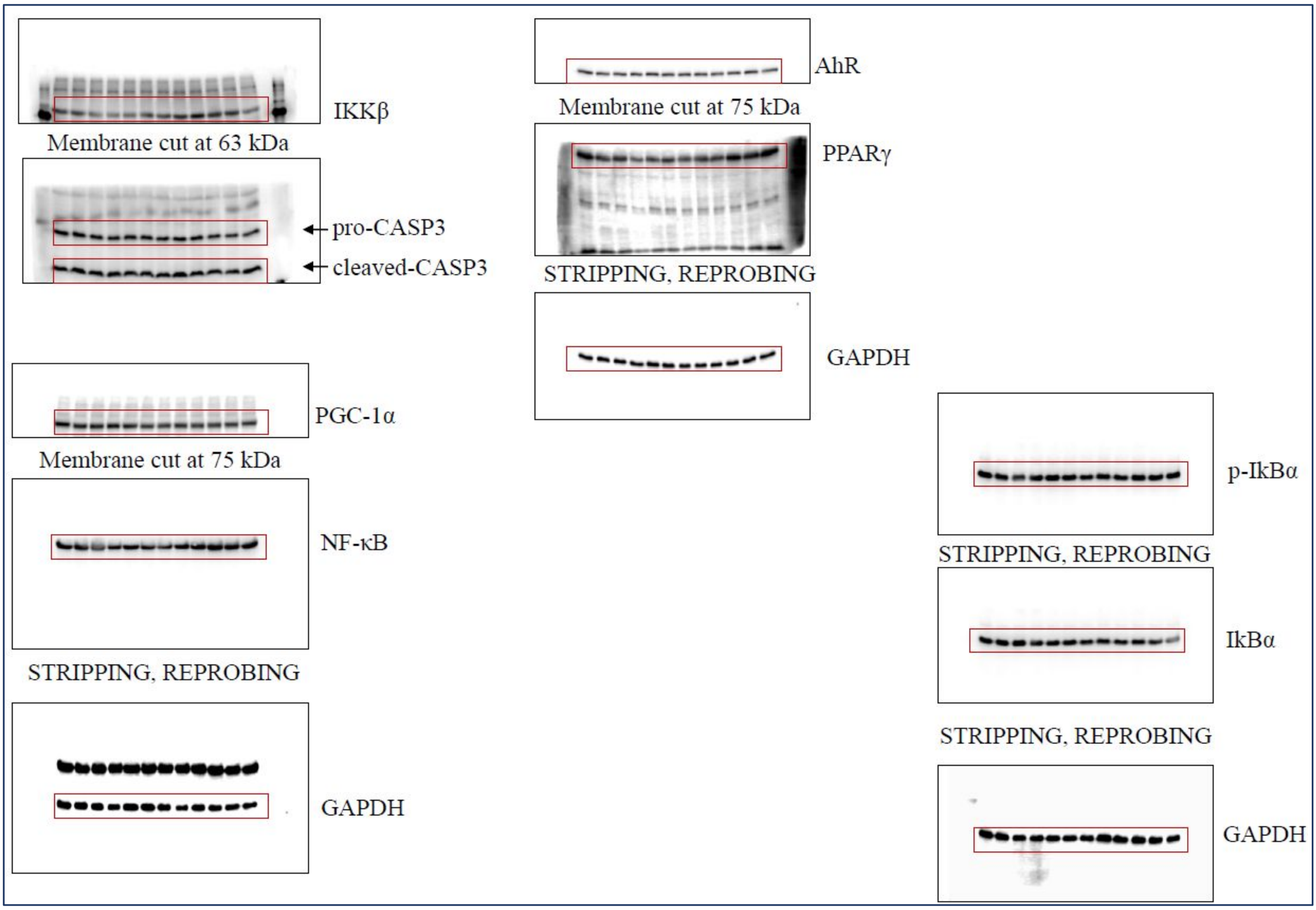

Figure S17. Raw blots

HOMO-LUMO DESCRIPTORS COMPUTATIONS FOR 3a–d

Table S1. The HOMO–LUMO descriptors of compounds **3a**–**d** computed using the IEFPCM solvation model (water as solvent); approximations: B3LYP/6-311++G(2d,3p)//B3LYP/6-31G(d,p).

Compound	HOMO Energy	LUMO Energy	HOMO-LUMO Gap	First Ionization Potential	Electron Affinity	Chemical Potential	Chemical Hardness	Electronegativity
**3c**	-6.57	-3.00	3.57	6.57	3.00	-4.78	1.79	4.78
**3d**	-6.70	-2.92	3.78	6.70	2.92	-4.81	1.89	4.81
**3a**	-6.33	-2.92	3.41	6.33	2.92	-4.62	1.70	4.62
**3b**	-6.33	-2.92	3.41	6.33	2.92	-4.62	1.70	4.62

Table S2. The HOMO–LUMO descriptors of compounds **3a**–**d** computed using the IEFPCM solvation model (water as solvent); approximations: CAM-B3LYP/6-311++G(2d,3p)//CAM-B3LYP/6-31G(d,p).

Compound	HOMO Energy	LUMO Energy	HOMO-LUMO Gap	First Ionization Potential	Electron Affinity	Chemical Potential	Chemical Hardness	Electronegativity
**3c**	-7.92	-1.81	6.11	7.92	1.81	-4.87	3.06	4.87
**3d**	-8.06	-1.72	6.35	8.06	1.72	-4.89	3.17	4.89
**3a**	-7.70	-1.72	5.98	7.70	1.72	-4.71	2.99	4.71
**3b**	-7.70	-1.72	5.98	7.70	1.72	-4.71	2.99	4.71

Table S3. The HOMO–LUMO descriptors of compounds **3a**–**d** computed using the IEFPCM solvation model (water as solvent); approximations: PW6B95D3/6-311++G(2d,3p)//PW6B95D3/6-31G(d,p).

Compound	HOMO Energy	LUMO Energy	HOMO-LUMO Gap	First Ionization Potential	Electron Affinity	Chemical Potential	Chemical Hardness	Electronegativity
**3c**	-6.86	-2.74	4.12	6.86	2.74	-4.80	2.06	4.80
**3d**	-6.99	-2.66	4.32	6.99	2.66	-4.82	2.16	4.82
**3a**	-6.61	-2.67	3.95	6.61	2.67	-4.64	1.97	4.64
**3b**	-6.61	-2.67	3.95	6.61	2.67	-4.64	1.97	4.64

Table S4. The HOMO–LUMO descriptors of compounds **3a**–**d** computed using the IEFPCM solvation model (water as solvent); approximations: APFD/6-311++G(2d,3p)//APFD/6-31G(d,p).

Compound	HOMO Energy	LUMO Energy	HOMO-LUMO Gap	First Ionization Potential	Electron Affinity	Chemical Potential	Chemical Hardness	Electronegativity
**3c**	-6.71	-2.92	3.78	6.71	2.92	-4.82	1.89	4.82
**3d**	-6.84	-2.84	4.00	6.84	2.84	-4.84	2.00	4.84
**3a**	-6.45	-2.84	3.62	6.45	2.84	-4.65	1.81	4.65
**3b**	-6.46	-2.84	3.62	6.46	2.84	-4.65	1.81	4.65

Table S5. The HOMO–LUMO descriptors of compounds **3a**–**d** computed using the IEFPCM solvation model (water as solvent); approximations: M062X/6-311++G(2d,3p)//M062X/6-31G(d,p).

Compound	HOMO Energy	LUMO Energy	HOMO-LUMO Gap	First Ionization Potential	Electron Affinity	Chemical Potential	Chemical Hardness	Electronegativity
**3c**	-7.88	-2.10	5.78	7.88	2.10	-4.99	2.89	4.99
**3d**	-8.01	-1.99	6.02	8.01	1.99	-5.00	3.01	5.00
**3a**	-7.66	-2.00	5.65	7.66	2.00	-4.83	2.83	4.83
**3b**	-7.66	-2.00	5.65	7.66	2.00	-4.83	2.83	4.83
